# Ultrasound targeted fatty acid nitroalkene-containing lipid nanoparticles attenuate inflammation and reverse myocardial fibrosis

**DOI:** 10.7150/thno.126503

**Published:** 2026-05-29

**Authors:** Muhammad W. Amjad, Soheb A. Mohammed, Marco Fazzari, Xucai Chen, Bruce A. Freeman, Terry O. Matsunaga, Yijen L. Wu, Sean Hartwick, Thomas Becker-Szurszewski, Devin R. E. Cortes, John J. Pacella

**Affiliations:** 1 Center for Ultrasound Molecular Imaging and Therapeutics, Heart and Vascular Medicine Institute, University of Pittsburgh; Pittsburgh, PA, USA.; 2 Department of Pharmacology and Chemical Biology, School of Medicine, University of Pittsburgh; Pittsburgh, PA, USA.; 3 Department of Biomedical Engineering and Department of Neurosurgery, University of Arizona; Tucson, AZ, USA.; 4 Department of Bioengineering, Swanson School of Engineering, University of Pittsburgh, Pittsburgh, PA, USA.; 5 Department of Pediatrics, University of Pittsburgh, Pittsburgh, PA, USA.; 6 Rangos Research Center Animal Imaging Core, Children's Hospital of Pittsburgh, Pittsburgh, PA, USA.

**Keywords:** fatty acid nitroalkenes, myocardial ischemia-reperfusion injury, myocardial fibrosis, lipid nanoparticles, ultrasound-targeted cavitation

## Abstract

**Rationale:**

Myocardial ischemia-reperfusion injury (MIRI) induces oxidative stress and inflammatory signaling that drive fibroblast activation, myocardial fibrosis, and progressive cardiac dysfunction, for which effective targeted therapies remain limited. The electrophilic fatty acid nitroalkene 10-nitro-octadec-9-enoic acid (NO₂-FA) exhibits anti-inflammatory and antifibrotic properties but pharmacological actions have been limited by extents of myocardial delivery. Ultrasound-targeted cavitation (UTC) using lipid-shelled gas-filled nanoparticles (LNPs) enables spatially controlled drug release and represents a promising theranostic strategy.

**Methods:**

NO₂-FA were incorporated in LNPs and delivered focally to the myocardium using UTC. Therapeutic efficacy was evaluated in rodent models of MIRI and myocardial fibrosis. In the MIRI model, animals received UTC + NO₂-FA LNPs, intravenous NO₂-FA, or sham treatment. Cardiac structure, function, and molecular remodeling were assessed using echocardiography, histological staining, polymerase chain reaction, and enzyme-linked immunosorbent assay. In the myocardial fibrosis model, additional analyses included immunohistochemistry and cardiovascular magnetic resonance imaging.

**Results:**

UTC-mediated delivery of NO₂-FA LNPs significantly reduced myocardial fibrosis compared with intravenous NO₂-FA (p = 0.03) and sham treatment (p = 0.001), as demonstrated by histological quantification and reduced late gadolinium enhancement. Targeted NO₂-FA delivery also improved cardiac output and favorably modulated molecular markers associated with fibrosis and inflammation. Across both experimental models, UTC-facilitated NO₂-FA delivery consistently demonstrated superior therapeutic efficacy relative to non-targeted administration.

**Conclusions:**

Spatially targeted delivery of NO₂-FA using ultrasound-mediated cavitation enhances cardioprotective and antifibrotic effects in experimental models of MIRI and myocardial fibrosis. This platform integrates targeted therapy with imaging-based assessment and supports further development of UTC-enabled theranostic approaches for ischemic heart disease.

## Introduction

Myocardial infarction (MI) remains a leading cause of morbidity and mortality worldwide and is a major contributor to the development of heart failure (HF) 1. Due to the limited regenerative capacity of the adult heart, MI results in substantial cardiomyocyte (CM) loss and excessive extracellular matrix (ECM) deposition processes collectively referred to as adverse cardiac remodeling. Cardiac fibrosis is a key pathological feature of this remodeling response 2-4. Therapeutic ultrasound (US) has emerged as a rapidly advancing strategy for enhancing reperfusion and enabling targeted drug delivery. Both our group and others have demonstrated that ultrasound-targeted cavitation (UTC) of microbubbles and nanoparticles effectively reduces microvascular obstruction (MVO) and improves tissue perfusion 5, 6. This approach uses image-guided ultrasound to induce cavitation of intravenously injected lipid nanoparticles (LNPs), generating intravascular shear forces. UTC not only restores perfusion by disrupting microthrombi but also activates endothelial nitric oxide (NO) signaling for local vasodilation 6-8. Importantly, UTC enables targeted therapeutic delivery, promoting site-specific accumulation of payloads at infarct regions, enhancing efficacy while minimizing off-target effects 9-13. Ultrasound-mediated sonoporation further facilitates intracellular uptake by transiently increasing cardiomyocyte membrane permeability.

Nitro-fatty acids are endogenous electrophilic lipids that covalently modify cysteine residues on ~100-150 proteins, regulating redox-sensitive signaling pathways including NF-κB, Keap1/Nrf2, and PPARγ 14-23. These modifications suppress inflammation, oxidative stress, and pathogenic activation of platelets, neutrophils, and macrophages, while limiting fibrosis and preserving endothelial function 24-31. Nitro-fatty acids also promote macrophage polarization toward a reparative M2 phenotype and have a favorable safety profile. Collectively, these properties make them uniquely suited for targeted delivery via LNPs, where their lipophilicity and stability allow controlled, focal release at sites of injury. Previous studies demonstrated that UTC-facilitated LNP delivery achieves preferential accumulation in ischemic tissue compared to remote sites, as quantified by LC-MS 37, supporting both the specificity and mechanistic rationale for focal therapy.

Herein, we report the anti-inflammatory, antifibrotic, and cardioprotective effects of UTC-mediated delivery of nitro-fatty acid in rodent models of myocardial ischemia-reperfusion injury (IRI) and fibrosis. 10-nitro-octadec-9-enoic acid (abbreviated as NO₂-FA throughout the manuscript) was selected based on its FDA IND approval and use in ongoing Phase 2 clinical trials for obesity-related asthma (NCT03762395). We hypothesized that NO₂-FA delivered via UTC-LNPs would enhance local drug accumulation, supplement UTC’s thrombolytic effects, and provide focal therapy to reduce fibrosis and adverse remodeling post-MI. Previously, NO₂-FA LNPs were tested in hindlimb ischemia-reperfusion, where ultrasound-induced oscillation and collapse of LNPs generated shear stress and microjets, enhancing clot fragmentation, permeability, and drug delivery 37. Extending this approach to cardiac models aims to evaluate both mechanistic efficacy and translational potential in disease-relevant contexts.

These strategies align with emerging multifunctional biomaterial approaches, such as hydrogel-based platforms that integrate regenerative, sensing, and therapeutic functions for myocardial repair. By combining targeted NO₂-FA delivery with ultrasound-mediated control, our approach achieves localized, responsive therapy while modulating pathological remodeling.

## Methods

### Study design

The goal of this study was to develop NO_2_-FA LNPs and investigate their cardioprotective efficacy in combination with UTC on novel rodent models of IRI and fibrosis. Male Wistar rats were used to develop robust MIRI and fibrosis models. Left anterior descending (LAD) coronary artery was completely occluded using a 6-0 suture for 60 min, followed by reperfusion. In MIRI experiment, rats were treated after LAD reperfusion, and the rats were survived after treatment for 2 h. In fibrosis experiment, rats were survived for 28 days post MIRI, followed by treatments (every alternate day) for 14 days. For UTC therapy, ultrasound was delivered using a single-element transducer operating at a center frequency of 1 MHz, driven by an arbitrary waveform generator coupled to a radiofrequency power amplifier. The transducer was positioned vertically over the left chest while the rat was maintained in the supine position, with the transducer placed approximately 1.5 cm above the chest surface. Acoustic coupling was achieved using ultrasound gel to ensure efficient energy transmission. Therapeutic ultrasound was administered as rapid, short pulses (center frequency 1 MHz; peak negative acoustic pressure 0.5 MPa; pulse duration 10 μs ON and 90 μs OFF; 50 pulses per burst; bursts repeated every 0.5 s). This pulsing strategy allowed replenishment of lipid nanoparticles (LNPs) within the myocardial microcirculation during treatment. Each treatment session lasted 15 min.

The outcome of UTC+NO_2_-FA LNPs treatment was measured using various techniques. Targeted delivery of NO_2_-FA using UTC+NO_2_-FA LNPs was quantified by extracting the tissue, pulverizing it and measuring NO_2_-FA concentration using LCMS. Myocardial functional parameters were measured using ultrasound. B- and M-mode clips of heart were recorded at short axis view. In both MIRI and fibrosis experiments, M-mode US images were used to compare the myocardial contractility of both anterior and posterior walls at different phases. In MIRI study, EF and FS during ischemia and post treatment were calculated. In fibrosis experiment, M-mode pictures were used to calculate LV fractional area change (surrogate of ejection fraction) and B-mode clips were used to calculate wall motion score index. Infarct area in MIRI experiment was measured using Masson’s Trichrome staining. rtPCR was performed using myocardial tissue to measure the mRNA expression of pro-inflammatory genes after treatment. ELISAs were performed using sera extracted from rats before euthanasia to quantify myocardial injury markers including troponin, LDH, myoglobin and CPK in both MIRI and fibrosis experiments. In the fibrosis experiment, short axis myocardial sections were stained with Masson’s Trichrome and H&E to visualize fibrosis and neutrophil infiltration and subsequent quantification as % area of section using ImageJ software. IHC staining was also performed to visualize collagen 1, collagen 3 and α-SMA biomarkers on myocardial sections followed by their quantification as % area of section using ImageJ software. In a separate cohort of rats, Evans blue/TTC staining of the myocardial sections was performed at the end of the experiment to visualize infarct area, followed by quantification (% infarct) of the myocardial section. Another set of rats was used to record CMR at the end of the experiment. LGE, cardiac output, ejection fraction, longitudinal, radial and circumferential strain, and extracellular volume (ECV) were determined. All quantitative assays were performed with a minimum of three technical replicates. The number of biological replicates for each experiment is specified in the corresponding figure legends. All animal procedures were carried out in compliance with institutional guidelines for the care and use of laboratory animals (IACUC protocol #22112218). A comprehensive description of the experimental procedures is provided in the Supplementary Materials.

## Results

### Fabrication of NO_2_-FA-lipid nanoparticles (NO_2_-FA LNPs)

**Figure 1:**
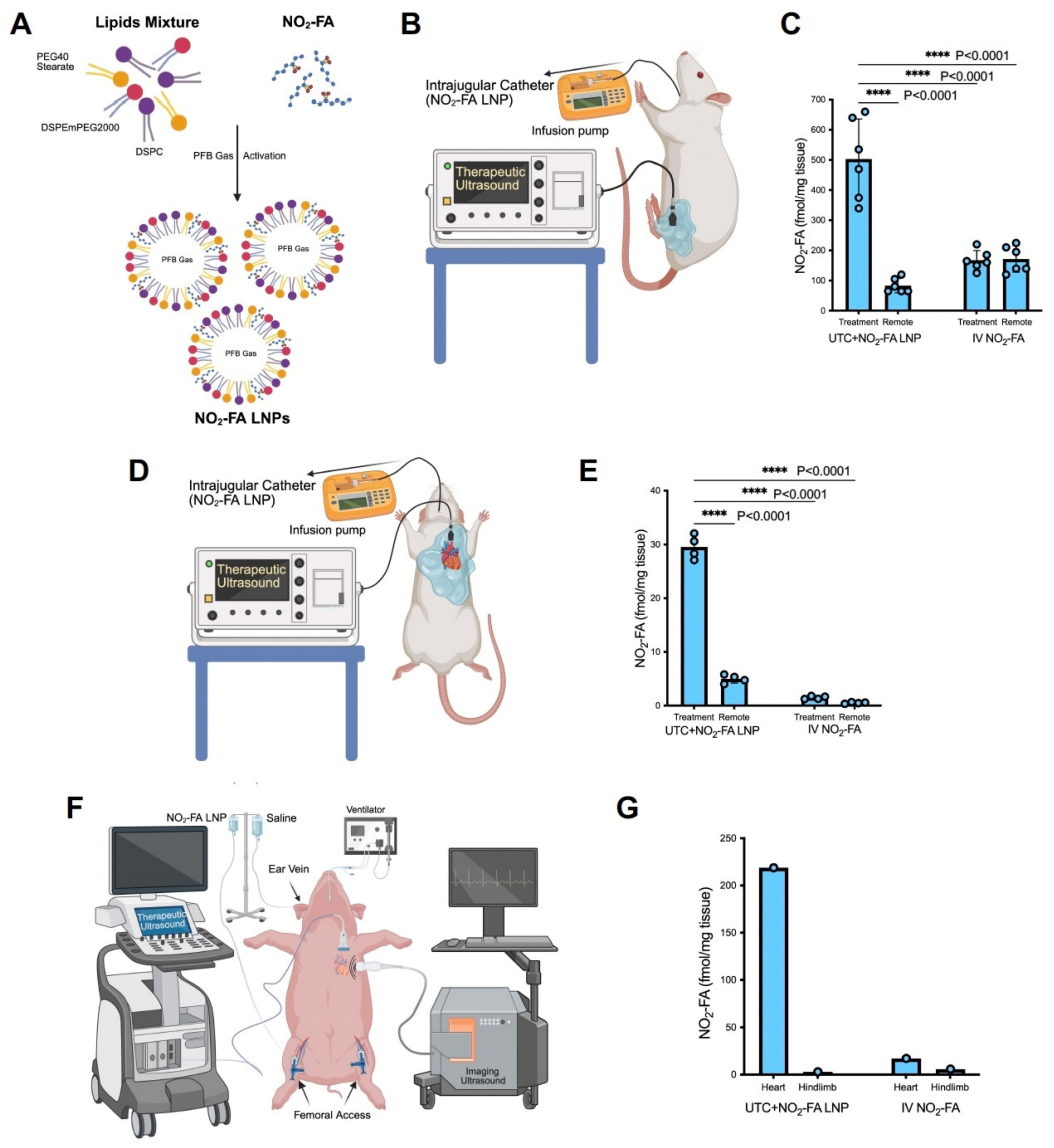
** Targeted delivery of NO_2_-FA by UTC+NO_2_-FA LNPs to the target sites (heart and hindlimb) in rodent and porcine models. A** Fabrication of NO_2_-FA LNPs from lipids and NO_2_-FA. The PFB gas entrapped in the core enables the LNPs to cavitate when US is applied. **B** Experimental setup for the targeted delivery of NO_2_-FA to healthy hindlimb of rat using intrajugular venous infusion of NO_2_-FA LNPs and simultaneous application of external focused ultrasound over the target hindlimb. **C** Quantification of NO_2_-FA in gastrocnemius (treatment site) and quadriceps femoris (remote site) after UTC+NO_2_-FA LNP or IV NO_2_-FA administration. Data are mean ± SEM of 6 rats per group; one-way ANOVA with Tukey’s multiple comparisons. **D** Experimental setup for the targeted delivery of NO_2_-FA to rat heart after inducing ischemia reperfusion injury, using intrajugular venous infusion of NO_2_-FA LNPs and simultaneous application of external focused ultrasound over the heart. **E** Quantification of NO_2_-FA in myocardial tissue (treatment site) and quadriceps femoris (remote site) after UTC+NO_2_-FA LNP or IV NO_2_-FA administration. Data are mean ± SEM of 4 rats per group; one-way ANOVA with Tukey’s multiple comparisons. **F** Experimental setup for the targeted delivery of NO_2_-FA to healthy porcine heart using intra-femoral venous infusion of NO_2_-FA LNPs and simultaneous application of external focused ultrasound over the heart. **G** Quantification of NO_2_-FA in myocardial tissue (treatment site) and quadriceps femoris (remote site) after UTC+NO_2_-FA LNP or IV NO_2_-FA administration. Data represent measurements from a single pig (n = 1).

The size distribution of NO_2_-FA LNPs was measured by Coulter counter analysis. The diameter of NO_2_-FA LNPs ranged between 1.2-3.0 μm, with the highest density of particles at 1.2 μm diameter, with a mean concentration of 3×10^9^ LNPs/mL. Figure 1A is an illustration showing the fabrication of NO_2_-FA LNPs. The NO_2_-FA content bound to NO_2_-FA-LNPs was measured using LC-MS. The average NO_2_-FA concentration was 260 μg per 3×10^9^ LNPs (in 1 mL), after subtraction of the subnatant containing NO_2_-FA that was not LNP-associated. One dose (1 mL) of NO_2_-FA LNP was infused over 15 min for UTMC treatment and controlled for with an equivalent IV dose of ‘free’ NO_2_-FA.

### Targeted delivery of NO_2_-FA to hindlimb and heart by UTC+NO_2_-FA LNP in rodent and porcine models

NO_2_-FA was delivered focally to the healthy hindlimb of rat using UTC+NO_2_-FA LNP (Fig. 1B). Figure 1B shows the experimental setup. The concentration of NO_2_-FA in targeted (healthy hindlimb tissue, gastrocnemius) and a remote site (quadriceps femoris on the ipsilateral hindlimb), treated with either UTC+NO_2_-FA LNPs or IV NO_2_-FA was quantified (Figure 1C). In UTC+NO_2_-FA LNP treatment, the concentration of NO_2_-FA in the targeted gastrocnemius muscle was 6-fold higher (503.3 fmol/mg) than in the remote untargeted quadriceps femoris (83.3 fmol/mg, *p <* 0.0001). Alternatively, the concentration of NO_2_-FA at the targeted and remote sites after IV ‘free’ NO_2_-FA treatment were similar (167.5 and 171.7 fmol/mg respectively), showing no indication of targeted delivery. Moreover, NO₂-FA concentration in the gastrocnemius after UTC+NO₂-FA LNP treatment was 4-fold higher than that present at either gastrocnemius or quadriceps femoris after systemic infusion of IV NO_2_-FA.

The experimental setup for the targeted delivery of NO_2_-FA to the rat heart following ischemia reperfusion injury is shown in Figure 1D. NO_2_-FA concentrations were quantified in heart and a remote site (quadriceps femoris on the ipsilateral hindlimb) after treatment with either UTC+NO_2_-FA LNPs or IV NO_2_-FA (Figure 1E). Following UTC+NO_2_-FA LNPs NO_2_-FA levels were significantly higher than at the remote site (29.5 and 5 fmol/mg respectively). In contrast, IV NO_2_-FA resulted in lower and comparable concentrations in heart and remote site (1.5 and 0.7 fmol/mg respectively).

NO_2_-FA delivery to the healthy porcine heart using UTC+NO_2_-FA LNPs (Figure 1F) gave 70-fold higher NO_2_-FA concentration in the heart than in the remote site (219 and 3 fmol/mg respectively) (Figure 1G). IV NO_2_-FA treatment resulted in an NO_2_-FA concentration in heart of 17.2 fmol/mg tissue while it was 5.9 fmol/mg tissue in remote site.

### Ischemia-reperfusion injury

#### The effects of UTC+NO_2_-FA LNP therapy on myocardial contractility, ejection fraction and fractional shortening

**Figure 2:**
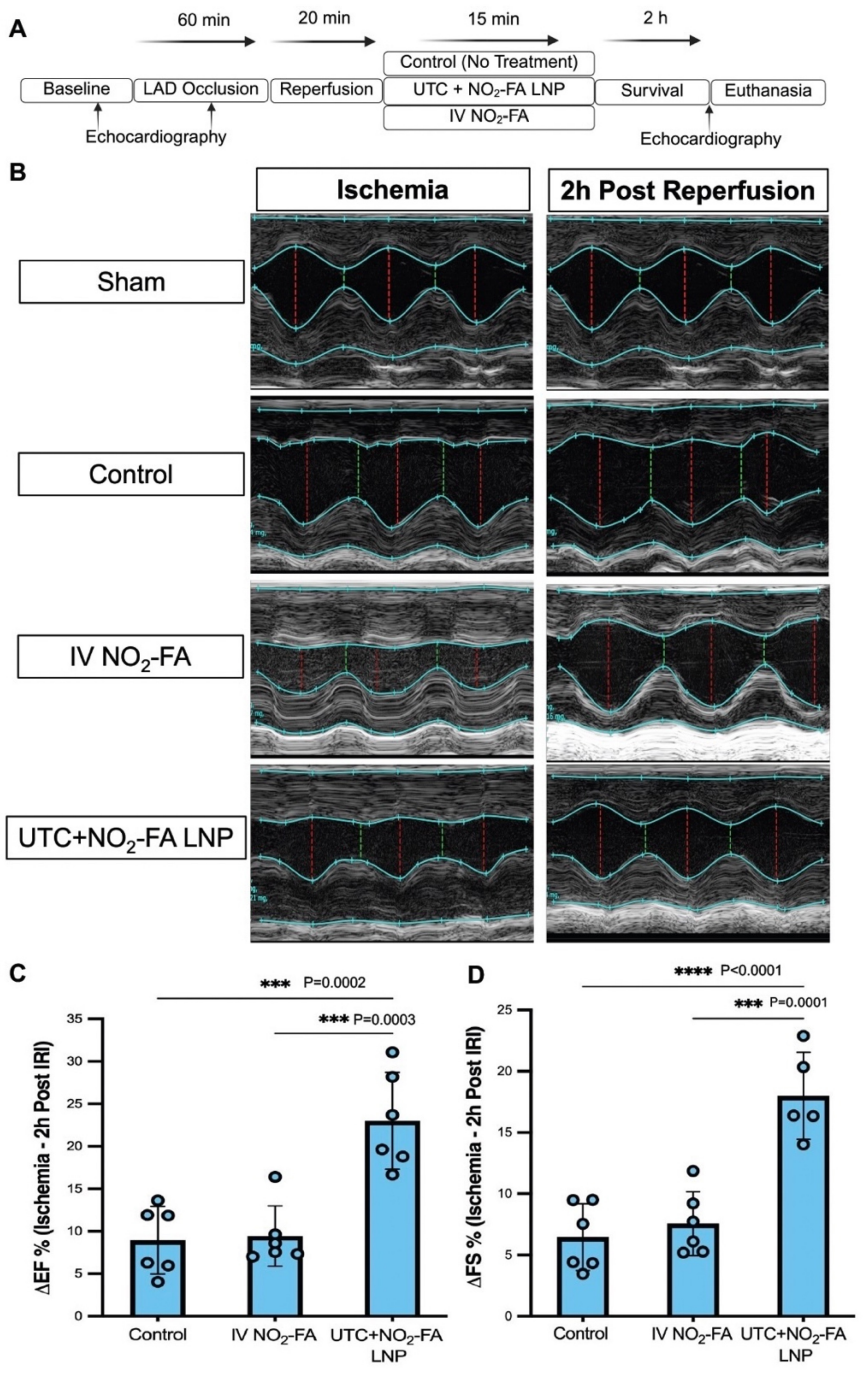
** UTC+NO_2_-FA LNPs significantly improve key cardiac function parameters post Ischemia Reperfusion Injury (IRI). A** Flow chart showing important steps of IRI experiment. **B** Representative M-mode ultrasound images of rat heart showing the movement of myocardium, recorded at 2 stages i.e. during ischemia and 2h post reperfusion from 4 groups (sham, control, IV NO_2_-FA only and UTC+ NO_2_-FA LNPs). Each image contains important information including ejection fraction (EF) %, fractional shortening (FR) %, internal diameter, heart rate (HR), stroke volume (SV), cardiac output (CO) and more. **C** Delta (Δ) EF % from during ischemia to 2h post reperfusion for control, IV NO_2_-FA only and UTC+NO_2_-FA LNPs groups. Data are mean ± SEM from 6 rats per group **D** Delta (Δ) FS % from during ischemia to 2h post reperfusion for control, IV NO_2_-FA only and UTC+NO_2_-FA LNPs groups. Data are mean ± SEM from 5-6 rats per group. Statistical analysis was performed by one-way ANOVA followed by Tukey's multiple comparisons test.

The efficacy of UTC+NO₂-FA LNP was evaluated in a rat model of myocardial ischemia-reperfusion (Figure 2A). Short-axis M-mode echocardiography images of the left ventricle were obtained at two key time points: during ischemia and 2 hours after reperfusion.

Representative images are shown in Figure 2B. In sham rats, contractility of both the anterior (top) and inferior (bottom) walls of the left ventricle was normal. In control rats subjected to ischemia, the left ventricular anterior wall became akinetic. Similarly, in the IV NO₂-FA treatment group, the anterior wall remained akinetic during ischemia. However, 2 hours after reperfusion and treatment, recovery of anterior wall contractility was improved compared to control. The most pronounced recovery of anterior wall motion at 2 hours post-reperfusion was observed in the UTC+NO₂-FA LNP group.

M-mode images were used to calculate the change in Ejection Fraction (ΔEF, %) (Figure 2C) and change in Fractional Shortening (ΔFS, %) (Figure 2D), defined as the improvement from ischemia to 2 hours post-reperfusion. For ΔEF, the control group showed a cumulative increase of 8.9 ± 3.982%. IV NO₂-FA treatment resulted in a similar increase of 9.4 ± 3.555% (ns, p = 0.98 vs control). In contrast, UTC+NO₂-FA LNP treatment produced a markedly greater improvement in EF (23.009 ± 5.698%), which was significantly higher than both control (p = 0.0002) and IV NO₂-FA (p = 0.0003). For ΔFS, the control group demonstrated a cumulative increase of 6.461 ± 2.727%. IV NO₂-FA treatment showed no significant change (7.567 ± 2.613%; ns, p = 0.80 vs control). Similar to EF, UTC+NO₂-FA LNP therapy resulted in the greatest improvement in FS (17.998 ± 3.555%), which was significantly greater than control (p < 0.0001) and IV NO₂-FA (p = 0.0001).

Together, these data demonstrate that UTC+NO₂-FA LNP treatment provides superior functional recovery following ischemia-reperfusion compared to systemic NO₂-FA administration or control treatment.

#### UTC+NO_2_-FA LNPs reduce infarct area, inflammation and myocardial injury

**Figure 3:**
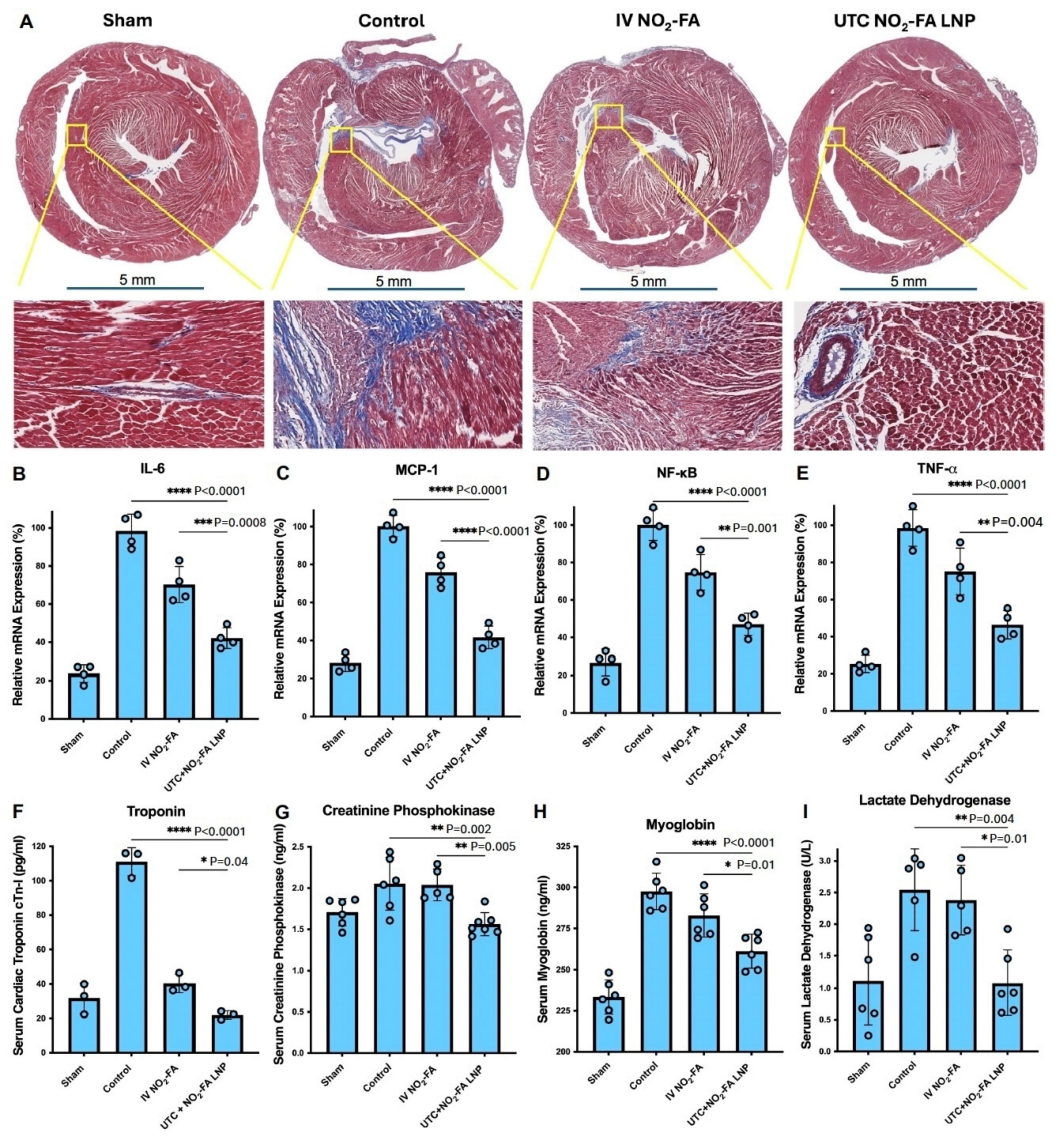
** UTC+NO_2_-FA LNPs reduce infarct size, ameliorate inflammation and myocardial injury post Ischemia Reperfusion Injury (IRI). A** Representative transverse section images of rat heart stained with Masson's trichrome. Area in the yellow box in each representative image is magnified at 12-15× below for better visualization.** B–E** Changes in mRNA expression of key inflammatory genes, including IL-6, MCP-1, NF-κB, and TNF-α, in myocardial tissue samples collected 2 h post-ischemia-reperfusion injury (IRI) from Sham, Control, IV NO₂-FA only, and UTC+NO₂-FA LNP groups (n = 4 per group). **F–I** Serum levels of myocardial injury and inflammation biomarkers, including cardiac troponin, creatinine phosphokinase, myoglobin, and lactate dehydrogenase, measured 2h post-IRI in Sham, Control, IV NO₂-FA only, and UTC+NO₂-FA LNP groups (n = 5–6 per group). All data are presented as mean ± SEM. Statistical significance in panels **B–I** was determined using one-way ANOVA followed by Tukey’s multiple comparisons test.

To visualize infarct size after ischemia-reperfusion injury (IRI) and subsequent treatment, histopathological analysis was performed. Representative short-axis myocardial sections stained with Masson’s Trichrome are shown in Figure 3A. The infarct area in the septal region is outlined in yellow and magnified.

In sham sections, no Masson’s Trichrome staining was observed. In contrast, control sections showed dense blue staining (fibrotic tissue) in the septum, extending into a significant portion of the antero-septum. IV NO₂-FA treatment reduced both the size and intensity of the infarcted area, which was largely confined to the septum. The smallest residual infarct area was observed in the UTC+NO₂-FA LNP group, where only minimal blue staining remained.

To assess inflammatory gene expression 2 h post reperfusion, qPCR analysis was performed for IL-6, MCP-1, NF-κB, and TNF-α (Figure 3B-E). IL-6 expression (Figure 3B) increased from 23.65 ± 4.47% in sham to 98.5 ± 8.85% in control. IV NO₂-FA reduced IL-6 to 70.25 ± 9.54% (p = 0.0008 vs control), while UTC+NO₂-FA LNPs further reduced IL-6 to 42.10 ± 5.41%, significantly lower than both control (p < 0.0001) and IV NO₂-FA (p = 0.0008). MCP-1 expression (Figure 3C) rose from 28.27 ± 4.496% in sham to 100.23 ± 5.750% in control. IV NO₂-FA reduced MCP-1 to 75.98 ± 7.676% (p = 0.0005), whereas UTC+NO₂-FA LNPs achieved the greatest reduction (41.742 ± 5.922%), significantly lower than both control and IV NO₂-FA (both p < 0.0001). NF-κB expression (Figure 3D) increased from 26.80 ± 6.959% in sham to 100.16 ± 8.336% in control. IV NO₂-FA reduced NF-κB to 74.71 ± 9.495% (p = 0.002), while UTC+NO₂-FA LNPs reduced it further to 47.008 ± 5.901% (p < 0.0001 vs control; p = 0.0015 vs IV). TNF-α expression (Figure 3E) increased from 25.33 ± 4.655% in sham to 98.56 ± 9.846% in control. IV NO₂-FA reduced TNF-α to 75.15 ± 12.577% (p = 0.016), whereas UTC+NO₂-FA LNPs reduced it to 46.49 ± 7.655% (p < 0.0001 vs control; p = 0.004 vs IV NO₂-FA).

Serum biomarkers of myocardial injury and inflammation were assessed by ELISA (Figure 3F–I). Troponin levels (Figure 3F) increased from 31.82 ± 8.815 pg/mL in sham to 111.1 ± 8.154 pg/mL in control. IV NO₂-FA reduced troponin to 40.41 ± 5.365 pg/mL (p < 0.0001), and UTC+NO₂-FA LNPs further reduced it to 22.04 ± 2.541 pg/mL (p < 0.0001 vs control; p = 0.04 vs IV). CPK levels (Figure 3G) increased from 1.71 ± 0.166 ng/mL in sham to 2.05 ± 0.319 ng/mL in control. IV NO₂-FA showed no significant change (2.04 ± 0.193 ng/mL), whereas UTC+NO₂-FA LNPs reduced CPK to 1.56 ± 0.139 ng/mL (p = 0.003 vs control; p = 0.006 vs IV). Myoglobin levels (Figure 3H) increased from 233.45 ± 10.391 ng/mL in sham to 297.59 ± 11.064 ng/mL in control. IV NO₂-FA did not significantly reduce myoglobin (283 ± 13.130 ng/mL; p = 0.20), whereas UTC+NO₂-FA LNPs reduced it to 261.24 ± 10.260 ng/mL (p = 0.0085 vs control). LDH levels (Figure 3I) increased from 1.118 ± 0.698 U/L in sham to 2.54 ± 0.649 U/L in control. IV NO₂-FA showed no significant reduction (2.383 ± 0.551 U/L), while UTC+NO₂-FA LNPs reduced LDH to 1.083 ± 0.510 U/L (p = 0.005 vs control; p = 0.01 vs IV NO₂-FA).

Overall, UTC+NO₂-FA LNP treatment consistently produced the greatest reduction in infarct size, inflammatory gene expression, and serum biomarkers of myocardial injury compared with control and IV NO₂-FA treatment.

#### The effect of UTC+NO_2_-FA LNP therapy on myocardial fibrosis

**Figure 4:**
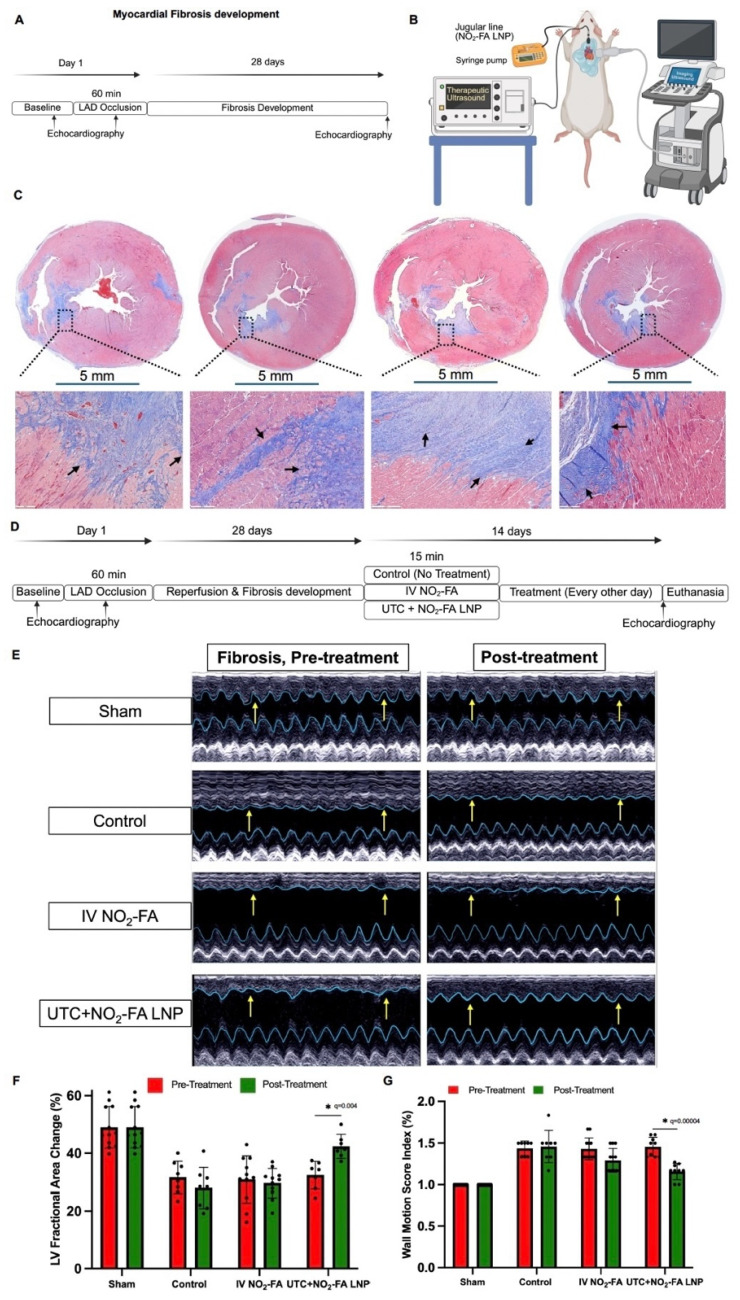
** UTC+NO_2_-FA LNPs significantly improve key cardiac function parameters post fibrosis. A** Flow chart showing important steps in the development of fibrosis. **B** Experimental setup for the treatment of myocardial fibrosis with IV NO_2_-FA and UTC+NO_2_-FA LNPs. **C** Development of rat myocardial fibrosis model. Transverse section images of rats (4) heart stained with Masson's trichrome taken at 28 days post IRI. Area in the black box in each representative image is magnified at 12-15× below for better visualization. **D** Flow chart showing important steps and timelines in the development of fibrosis and its treatment. **E** Representative M-mode ultrasound images of rat heart showing the movement of myocardium, recorded at 2 stages i.e. fibrosis, pre-treatment and post treatment.** F** Left ventricular (LV) fractional area change (%) measured at two stages-fibrosis (pre-treatment) and post-treatment in Sham, Control, IV NO₂-FA only, and UTC+NO₂-FA LNP groups (n = 7-12 per group). **G** Left ventricular wall motion score index (WMSI) measured at two stages-fibrosis (pre-treatment) and post-treatment in Sham, Control, IV NO₂-FA only, and UTC+NO₂-FA LNP groups (n = 7-12 per group). All data are presented as mean ± SEM. Statistical significance in panels **F–G** was determined using multiple unpaired t-tests.

We developed a rodent model of myocardial fibrosis to study the therapeutic potential of UTC+NO_2_-FA LNPs. Figure 4A-B. In a cohort of rats, baseline echocardiography measurements were recorded. To create a myocardial infarct and subsequent fibrosis, the chest was opened, and the LAD was occluded using a 6-0 suture for 60 min, followed by reperfusion. Rats were studied 28 days later for a drug impact on fibrosis. Echocardiography was repeated on day 28, which revealed a consistent decline in myocardial function in all IR rats. The rats were euthanized, hearts were harvested, transversely sectioned and stained with Masson's trichrome. Figure 4C shows Masson's trichrome stained sections (taken from the mid papillary muscle level of the LV myocardium). A consistent level of fibrosis was observed in the septal region of the left ventricle. The area outlined (rectangle with black dotted lines) is magnified below in each respective image, showing blue-stained fibrotic tissue as well as neighboring healthy tissue. These findings (decline in cardiac function and Masson's trichrome staining of heart) confirmed the consistent development of myocardial fibrosis.

#### UTC+NO_2_-FA LNP therapy improves myocardial contractility, fractional area change, and wall motion score indices in rat myocardial fibrosis

To evaluate the therapeutic effect of UTC+NO₂-FA LNPs on myocardial function, closed-chest echocardiography was performed before and after treatment. Both B-mode wall motion clips and M-mode still frames were recorded.

Figure 4D illustrates the key steps in the myocardial fibrosis study, including the treatment regimens. Representative M-mode images are shown in Figure 4E at two time points: baseline fibrosis before treatment (day 28 following LAD ligation) and post-treatment (day 42). The images show thickening of the left ventricular anterior and posterior walls, outlined in blue. At baseline (day 28), anterior wall hypo-contractility was evident in all groups (control, IV NO₂-FA, and UTC+NO₂-FA LNP), indicated by yellow arrows. After treatment, only a slight improvement in anterior wall contractility was observed in the IV NO₂-FA group, whereas the greatest recovery of anterior wall contractility occurred in the UTC+NO₂-FA LNP group. M-mode images were used to calculate cumulative left ventricular fractional area change (FAC), a surrogate measure of ejection fraction, at both time points (Figure 4F). In the control group, FAC declined from 31.6% at day 28 to 27.9% at day 42. IV NO₂-FA treatment did not significantly alter FAC (30.9% pre-treatment vs 29.6% post-treatment; q = 0.66). In contrast, UTC+NO₂-FA LNP treatment significantly increased FAC from 32.4% at baseline to 42.3% post-treatment (q = 0.004).

B-mode wall motion clips were used to calculate the left ventricular wall motion score index (WMSI) (Figure 4G). A WMSI of 1 represents normal function, with higher values indicating worse systolic performance. In the control group, WMSI showed no significant change (1.43 at day 28 vs 1.45 at day 42; ns). IV NO₂-FA treatment produced a modest, non-significant decrease in WMSI (1.43 pre-treatment to 1.29 post-treatment; q = 0.02). The greatest improvement was observed in the UTC+NO₂-FA LNP group, where WMSI decreased significantly from 1.45 to 1.16 (q = 0.00004).

Overall, UTC+NO₂-FA LNP treatment produced the most substantial improvement in myocardial performance, as demonstrated by enhanced M-mode contractility, increased LV FAC, and reduced WMSI compared with control and IV NO₂-FA treatment.

#### Reversal of fibrosis and reduction in neutrophil infiltration following treatment with UTC+NO_2_-FA LNPs

**Figure 5:**
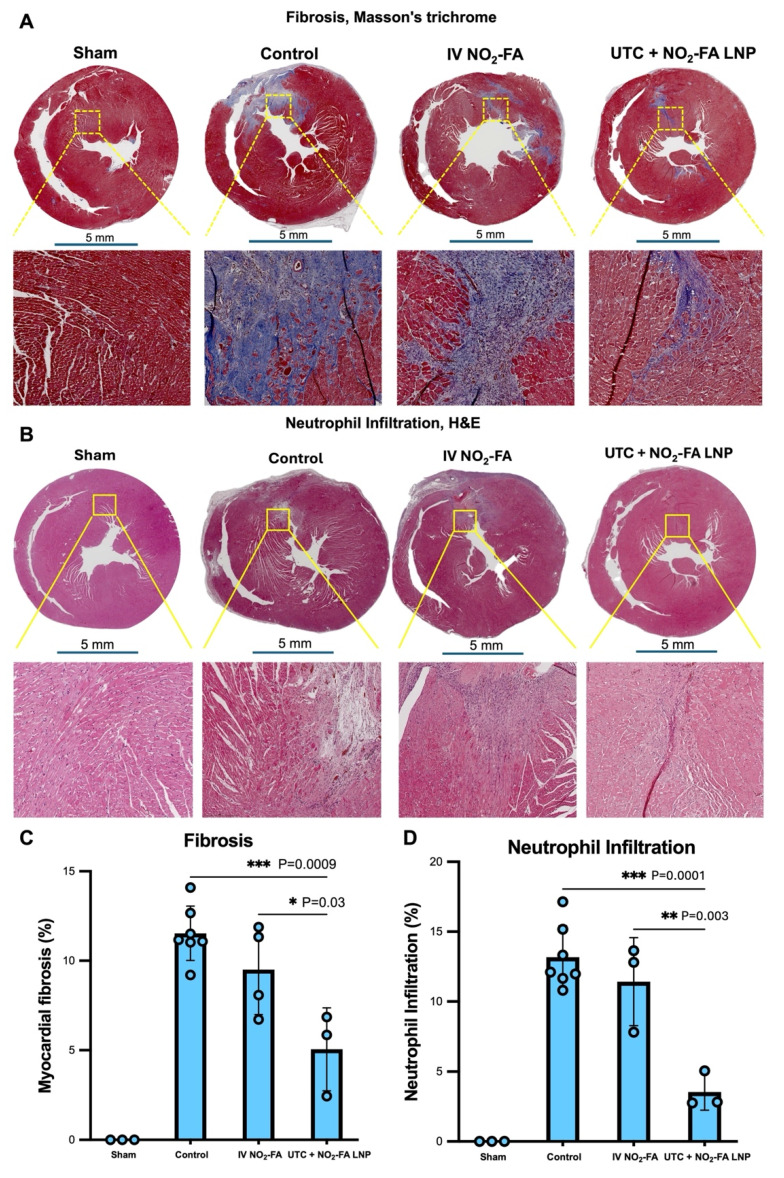
** UTC+NO_2_-FA LNPs reverse fibrosis and reduce neutrophil infiltration. A** Representative transverse section images of rat heart stained with Masson's trichrome. Area in the yellow box in each representative image is magnified at 12-15× below for better visualization. **B** Representative transverse section images of rat heart stained with H&E. Area in the yellow box in each representative image is magnified at 12-15× below for better visualization.** C** Cumulative myocardial fibrosis (%) in cardiac sections post-treatment (n = 3-7 per group). **D** Cumulative neutrophil infiltration (%) in cardiac sections post-treatment (n = 3–7 per group). All data in **C-D** are presented as mean ± SEM. Statistical significance was determined using one-way ANOVA followed by Tukey’s multiple comparisons test.

To study the effect of NO_2_-FA treatment on reversing myocardial fibrosis, we performed Masson's trichrome staining of the myocardial transverse sections taken at 42 days post IRI. Figure 5A shows representative myocardial section pictures from each group stained with Masson's trichrome (binds to collagen, characteristic blue color). As expected, sham rats did not exhibit Masson's trichrome staining, confirming normal healthy myocardium with no collagen deposition. The area of interest is highlighted and magnified with a square box outlined with a yellow dotted line. The control section exhibited a strong presence of Masson's trichrome stain in the anteroseptal region of the left ventricle with extension toward the right ventricle. In the magnified image, intense collagen deposition could be seen in the anteroseptal region of the left ventricle. Overall, we found decreased collagen deposition in the IV NO_2_-FA treatment group compared to controls. However, scattered trichrome staining is still present in the anterior, anterolateral and inferolateral regions of the LV. The greatest reduction in collagen deposition and fibrosis occurred following UTC+NO_2_-FA LNP treatment; in this group, the area of collagen deposition was markedly reduced, and it was restricted only to anterior and anteroseptal regions. In addition, the color intensity of the Masson’s trichrome stain was also reduced, indicating the areas of fibrosis were also less dense/concentrated.

To study myocardial neutrophil infiltration, we performed H&E staining of the myocardial transverse sections. Figure 5B shows representative images from sham, control, IV NO_2_-FA and UTC+NO_2_-FA LNP treatment groups. Sham and its magnified image showed healthy myocardium with no signs of neutrophil infiltration. In the control image, areas with intense neutrophil infiltration could be seen not only in anterior region but also in right ventricle, demonstrating a pervasive LAD infarction. Treatment with IV NO_2_-FA reduced the neutrophil infiltration however, it was still present in the anteroseptal region as well as the right ventricle. A more extensive reduction in both the area and intensity of neutrophil infiltration occurred after UTC+NO_2_-FA LNP treatment.

Masson’s Trichrome-stained myocardial transverse sections were used to quantify the percentage area of fibrosis within the left ventricle (LV) (Figure 5C). In the control group, fibrosis occupied 11.54 ± 1.520% of the LV. IV NO₂-FA treatment slightly reduced fibrosis to 9.50 ± 2.502%, but this reduction was not statistically significant (p = 0.32 vs control). The lowest level of fibrosis was observed in the UTC+NO₂-FA LNP group, where fibrosis was reduced to 5.05 ± 2.311% of the LV. This reduction was statistically significant compared to both control (p = 0.001) and IV NO₂-FA treatment (p = 0.03). H&E-stained myocardial sections were used to quantify neutrophil infiltration as a percentage of LV area (Figure 5D). In the control group, neutrophil infiltration affected 13.17 ± 2.248% of the LV. IV NO₂-FA treatment showed a similar level of infiltration (11.42 ± 3.149%), which was not significantly different from control (p = 0.63). In contrast, UTC+NO₂-FA LNP treatment markedly reduced neutrophil infiltration to 3.55 ± 1.304% of the LV area. This reduction was statistically significant compared to both control (p = 0.0001) and IV NO₂-FA treatment (p = 0.003). Overall, UTC+NO₂-FA LNP treatment resulted in the greatest reduction in both myocardial fibrosis and neutrophil infiltration compared with the other treatment groups.

#### UTC+NO_2_-FA LNPs reversed established fibrosis by reducing collagen 3, collagen 1, and α-SMA

To assess the presence and distribution of fibrotic proteins within myocardial tissue, immunohistochemistry (IHC) was performed on transverse heart sections.

**Figure 6:**
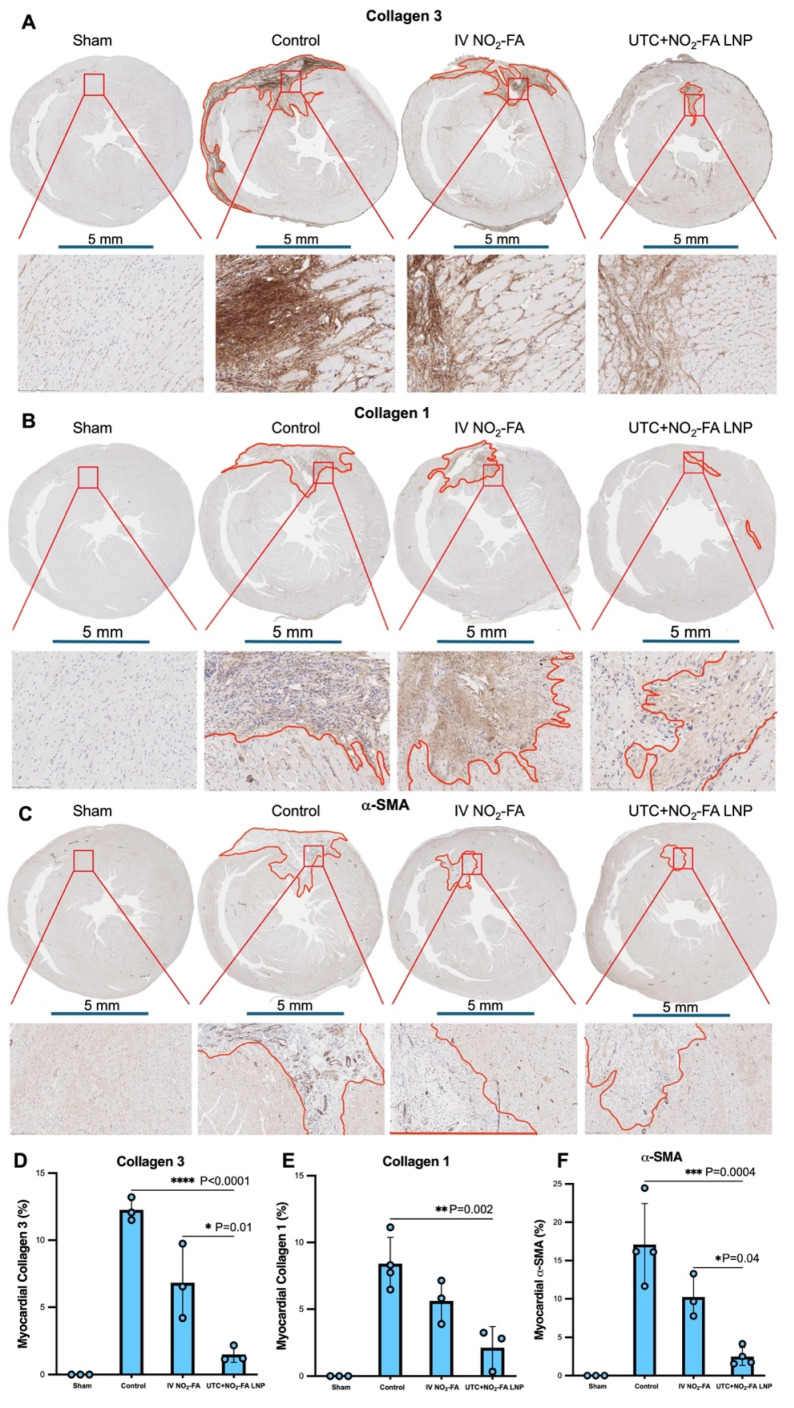
** UTC+NO_2_-FA LNPs significantly reduced fibrotic lesions as revealed by Immunohistochemistry (IHC). A-C** Representative transverse section images of rat heart stained with collagen 3 **A**, collagen 1 **B** and α-smooth muscle actin (SMA) **C** antibodies. Area in the red box in each representative image is magnified at 12-15× below for better visualization. **D** Cumulative myocardial collagen 3 (%) in the section post treatment (n = 3). **E** Cumulative myocardial collagen 1 (%) in the section post treatment (n = 3-4). **F** Cumulative myocardial α-SMA (%) in the section post treatment (n = 3-4). All data in **D–F** are presented as mean ± SEM. Statistical significance was determined using one-way ANOVA followed by Tukey’s multiple comparisons test.

Figure 6A shows representative images of collagen 3 staining. Collagen 3 is a marker of fibrosis and is used to monitor anti-fibrotic therapeutic effects. As expected, no collagen 3 staining was detected in sham sections. In control sections, intense brown staining indicating collagen 3 was observed in the left ventricular (LV) anteroseptum and right ventricle (RV). On magnification, collagen 3 staining was clearly distinguishable from healthy myocardium, and staining in the anteroseptum appeared darker and more intense than in the RV. In the IV NO₂-FA treated group, collagen 3 staining was limited to the anteroseptum and appeared smaller in area and lighter in intensity compared to control. The greatest reduction in both area and staining intensity was observed in the UTC+NO₂-FA LNP group. In this group, collagen 3 staining was minimal and largely confined to the anterior wall.

Figures 6B-C show representative images of collagen 1 and α-SMA staining. Like collagen 3, both collagen 1 and α-SMA are established markers of fibrosis and myofibroblast activation. Collagen 1 staining appeared light brown and was readily visible, whereas α-SMA staining required magnification for clear visualization. The highest levels of collagen 1 and α-SMA were observed in control sections. IV NO₂-FA treatment reduced the presence of both markers, while UTC+NO₂-FA LNP treatment resulted in the greatest reduction.

Quantification of IHC images was performed to calculate the percentage of myocardial tissue area positive for collagen 3, collagen 1, and α-SMA (Figures 6D-F). For collagen 3 (Figure 6D), 12.26 ± 0.866% of myocardial tissue was positive in control rats. IV NO₂-FA treatment significantly reduced this to 6.83 ± 2.781% (p = 0.0089). The lowest level of collagen 3 was observed in the UTC+NO₂-FA LNP group (1.51 ± 0.585%), which was significantly lower than both control (p < 0.0001) and IV NO₂-FA (p = 0.01). For collagen 1 (Figure 6E), 8.41 ± 1.967% of myocardial tissue was positive in control sections. IV NO₂-FA reduced this to 5.62 ± 1.625%, but the reduction was not statistically significant (p = 0.16). The lowest level of collagen 1 was observed after UTC+NO₂-FA LNP treatment (2.14 ± 1.578%), which was significantly lower than control (p = 0.002). For α-SMA (Figure 6F), 17.11 ± 5.344% of myocardial tissue was positive in control sections. IV NO₂-FA reduced α-SMA to 10.23 ± 2.802%, though this did not reach statistical significance (p = 0.08). The lowest α-SMA expression was observed in the UTC+NO₂-FA LNP group (2.496 ± 1.182%), which was significantly lower than both control (p = 0.0004) and IV NO₂-FA (p = 0.05).

Overall, UTC+NO₂-FA LNP treatment consistently produced the greatest reduction in collagen 3, collagen 1, and α-SMA expression, indicating superior attenuation of fibrotic remodeling compared to control and IV NO₂-FA treatment.

#### UTC+NO_2_-FA LNPs treatment markedly reduced myocardial infarct size, inflammation, and injury

**Figure 7:**
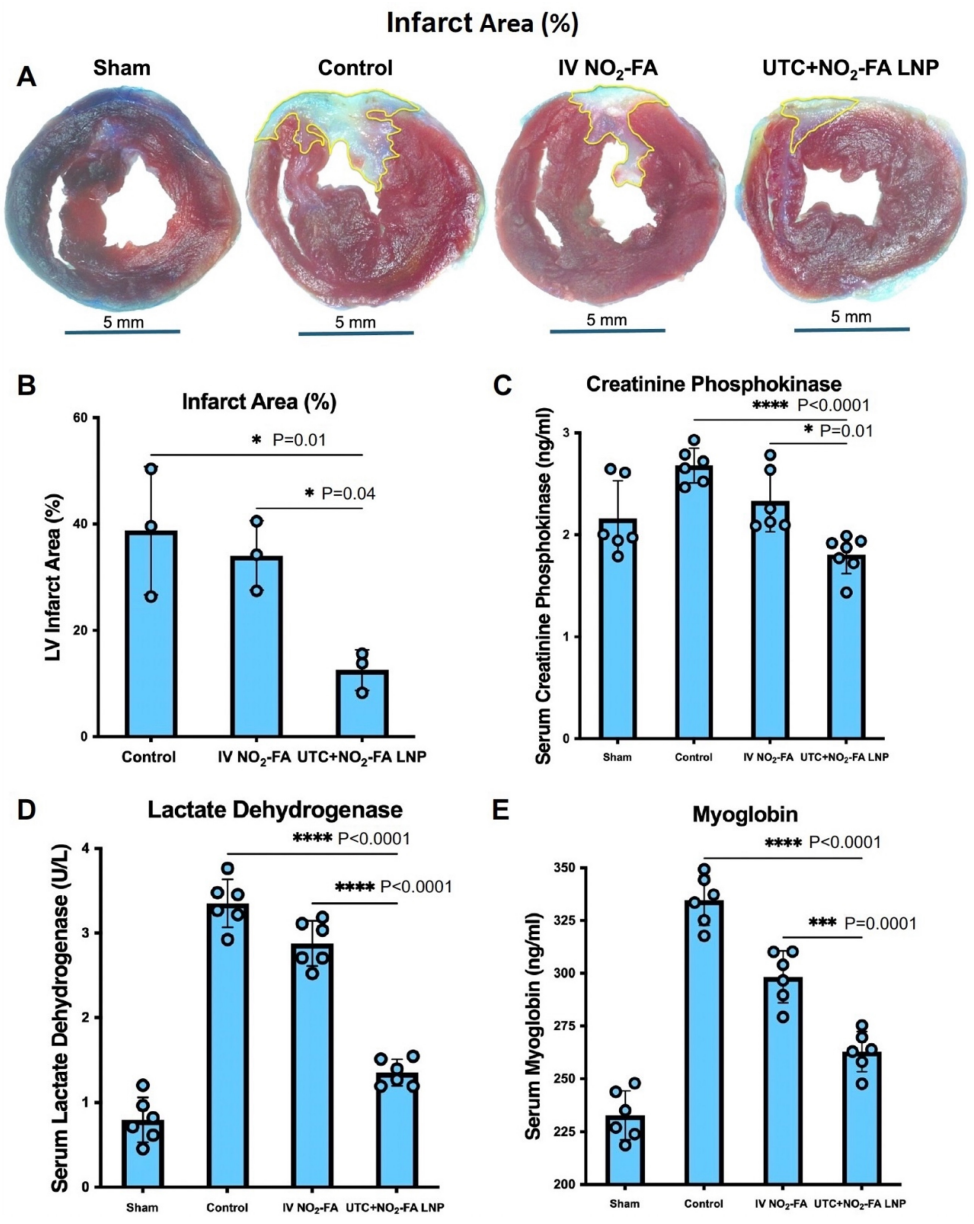
** UTC+NO_2_-FA LNPs reduce infarct and ameliorate inflammation post fibrosis. A** Representative transverse section images of rat heart stained with Evan’s blue and TTC. Area in the yellow box in each representative image shows the infarct area***.* B** Cumulative left ventricular (LV) infarct area (%) in cardiac sections post-treatment (n = 3 per group). **C-E** Serum levels of key biomarkers of myocardial injury, fibrosis, and inflammation measured 14 days post-treatment in Sham, Control, IV NO₂-FA only, and UTC+NO₂-FA LNP groups. **C** Creatinine phosphokinase (n = 6-7 per group). **D** Lactate dehydrogenase (n = 6 per group). **E** Myoglobin (n = 6 per group). All data in **B-E** are presented as mean ± SEM. Statistical significance was determined using one-way ANOVA followed by Tukey’s multiple comparisons test.

To visualize myocardial infarct size, Evan’s blue and TTC staining was performed (Figure 7A). After staining, viable tissue appears brick red/blue, whereas infarcted (non-viable) tissue appears white/grey.

In sham rats, myocardial sections appeared uniformly brick red with a slight blue tint, consistent with healthy myocardium. In control rats, sections from the anteroseptum and adjacent right ventricle showed prominent white/grey regions, indicating infarcted tissue. IV NO₂-FA treatment reduced the infarct size, which was largely confined to the anteroseptum. The smallest infarct area was observed in the UTC+NO₂-FA LNP group. Quantification of infarct area is shown in Figure 7B. In control rats, 38.7% of myocardial tissue was infarcted. IV NO₂-FA treatment reduced infarct size to 34.0%, but this reduction was not statistically significant (p = 0.77). In contrast, UTC+NO₂-FA LNP treatment reduced infarct area to 12.5%, which was significantly lower than both control (p = 0.02) and IV NO₂-FA treatment (p = 0.04).

To further assess myocardial injury and inflammation, ELISA was performed on serum collected before euthanasia to measure creatinine phosphokinase (CPK), lactate dehydrogenase (LDH), and myoglobin (Fig. 7C-E), all established markers of myocardial injury. CPK levels (Figure 7C) were 2.1 ng/mL in healthy (sham) rats. Levels increased to 2.7 ng/mL in control rats. IV NO₂-FA reduced CPK to 2.3 ng/mL, but this change was not statistically significant (p = 0.14). UTC+NO₂-FA LNP treatment produced the greatest reduction, lowering CPK to 1.8 ng/mL, which was significantly lower than control (p < 0.0001) and IV NO₂-FA (p = 0.01). LDH levels (Figure 7D) were 0.74 U/L in healthy rats and increased to 3.2 U/L in control rats. IV NO₂-FA treatment significantly reduced LDH to 2.98 U/L (p = 0.017). UTC+NO₂-FA LNP treatment further reduced LDH to 1.22 U/L, which was significantly lower than both control and IV NO₂-FA (both p < 0.0001). Myoglobin levels (Figure 7E) were 232.7 ng/mL in healthy rats and increased to 334.5 ng/mL in control rats. IV NO₂-FA significantly reduced myoglobin to 298.5 ng/mL (p = 0.0001). UTC+NO₂-FA LNP treatment further reduced myoglobin to 263 ng/mL, which was significantly lower than control (p < 0.0001) and IV NO₂-FA (p = 0.0001).

Overall, UTC+NO₂-FA LNP treatment consistently resulted in the greatest reduction in infarct size and serum biomarkers of myocardial injury compared with control and IV NO₂-FA treatment.

#### Treatment with UTC+NO_2_-FA LNPs reduced infarct volume and improved cardiac output on cardiovascular magnetic resonance (CMR) imaging

To evaluate fibrosis and myocardial function, cardiac magnetic resonance (CMR) imaging was performed before euthanasia.

**Figure 8:**
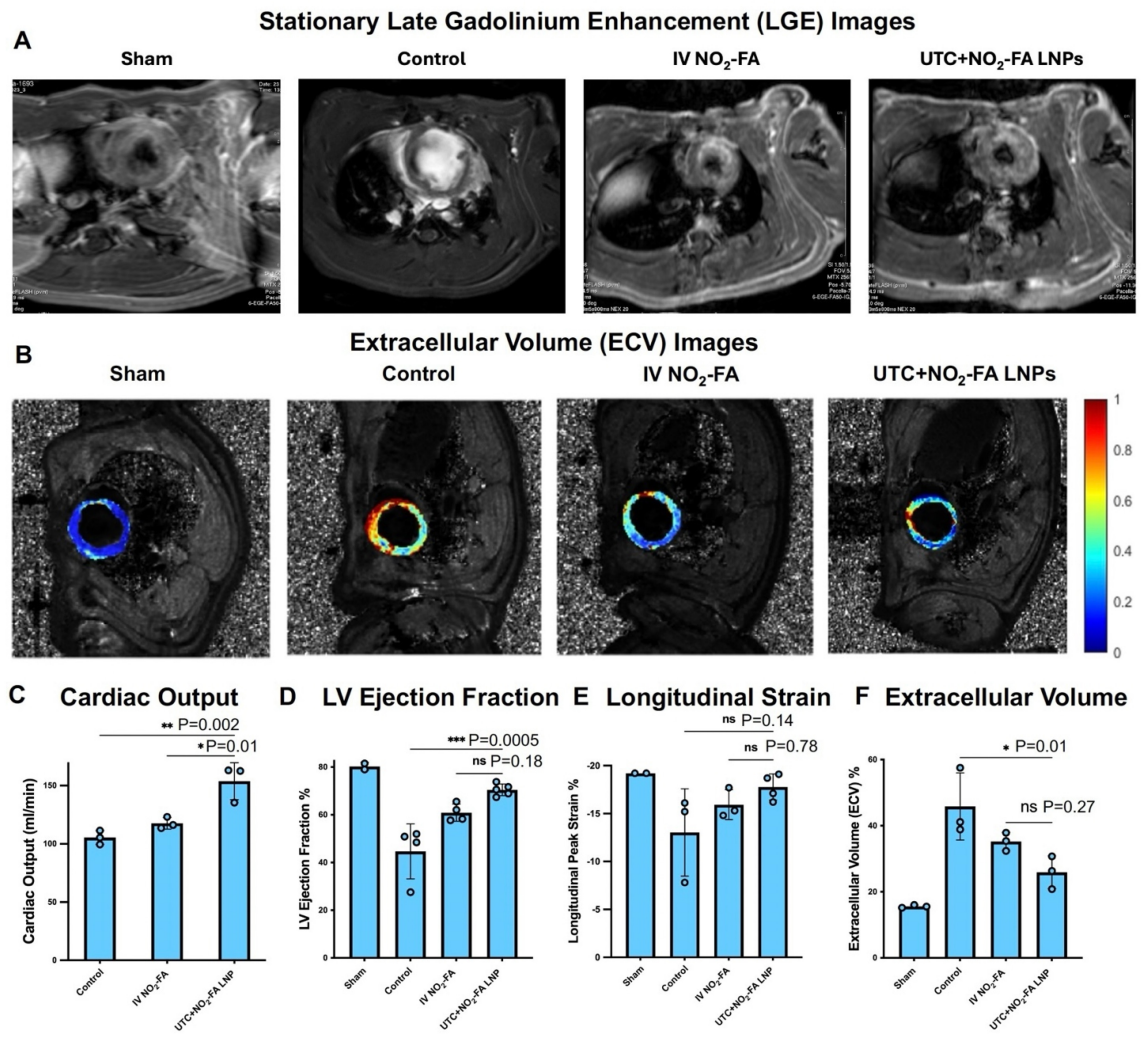
** UTC+NO_2_-FA LNPs treatment reduced LGE and ECV (%), enhanced cardiac output and improved longitudinal strain post fibrosis. A** Representative LGE images of the heart. Bright white areas in the myocardium show fibrosis. **B** Representative ECV images of the heart. Blue color in the circle shows less ECV while red patches show high ECV. **C** Cumulative cardiac output (mean ± SEM), calculated as stroke volume × heart rate, for control, IV NO₂-FA, and UTC+NO₂-FA LNPs groups (n = 3). **D** Cumulative left ventricular ejection fraction (LV EF, mean ± SEM) at the end of the experiment (n = 2-5). **E** Cumulative longitudinal strain (mean ± SEM) at the end of the experiment (n = 2-4). **F** Cumulative extracellular volume (ECV, %, mean ± SEM) measured prior to euthanasia (n = 3). Data are presented as mean ± SEM. Statistical significance was determined using one-way ANOVA followed by Tukey’s multiple comparisons test.

Figure 8A shows representative short-axis late gadolinium enhancement (LGE) images. Areas of fibrosis appear white due to gadolinium enhancement. As expected, sham hearts showed no LGE, indicating normal myocardium. In contrast, control hearts exhibited marked LGE in the myocardium, consistent with significant fibrosis. IV NO₂-FA treatment was associated with a visible reduction in LGE, indicating reduced fibrosis. The greatest reduction was observed in the UTC+NO₂-FA LNP group, where myocardial fibrosis appeared substantially lower than both control and IV NO₂-FA groups.

Figure 8B shows representative extracellular volume (ECV) maps, which paralleled the LGE findings. ECV was highest in controls and lowest in the UTC+NO₂-FA LNP group, indicating reduced fibrotic remodeling with treatment.

Cardiac output (CO) was calculated from CMR by multiplying stroke volume by heart rate (Figure 8C). In control rats, cumulative CO was 105.4 mL/min. IV NO₂-FA treatment increased CO numerically to 117.6 mL/min, but this was not statistically significant (p = 0.37). UTC+NO₂-FA LNP treatment produced the greatest improvement, increasing CO to 153.7 mL/min, which was significantly higher than both control (p = 0.003) and IV NO₂-FA (p = 0.01).

Ejection fraction (EF) measured by CMR is shown in Figure 8D and mirrored the ΔEF findings obtained by ultrasound. Sham rats had an EF of 80 ± 1.75%. EF in control rats was reduced to 44.7%. UTC+NO₂-FA LNP treatment significantly improved EF to 70.5%, compared with control (p = 0.0005). EF in the IV NO₂-FA group was 60.9%, which was numerically higher than control but did not reach statistical significance compared to UTC+NO₂-FA LNP (p = 0.18).

Figure 8E shows longitudinal strain. Sham rats exhibited a strain value of -19.2%. This was reduced to -13.03% in controls, indicating impaired function. IV NO₂-FA treatment improved strain numerically to -15.9%, but this did not reach statistical significance (p = 0.53). UTC+NO₂-FA LNP treatment showed the greatest improvement, restoring strain toward sham levels (-17.8%), although this did not reach statistical significance (p = 0.78).

Quantified ECV (%) is shown in Figure 8F. ECV was 15.5% in sham rats and increased to 45.8% in controls. IV NO₂-FA treatment reduced ECV numerically to 35.2%, but this did not reach statistical significance (p = 0.19). UTC+NO₂-FA LNP treatment reduced ECV to 25.9%, which was significantly lower than control (p = 0.013) and numerically lower than IV NO₂-FA.

Overall, CMR imaging demonstrated that UTC+NO₂-FA LNP treatment produced the greatest improvement in myocardial fibrosis and functional parameters, including cardiac output, ejection fraction, longitudinal strain, and extracellular volume, compared with control and IV NO₂-FA treatment.

## Discussion

This study reveals that NO_2_-FA can be effectively incorporated into lipid nanoparticles and locally delivered to rat and porcine heart via ultrasound-targeted cavitation (UTC), providing a controlled targeted delivery strategy of a therapeutic electrophilic lipid mediator. Compared with systemic administration of free NO₂-FA, UTC+NO_2_-FA LNPs significantly improved contractility, enhanced EF and FS, and reduced infarct size, pro-inflammatory marker expression and indices of myocardial injury in the IRI model. Similar therapeutic effects were observed in onset of myocardial fibrosis in a chronic IR model, including reduced fibrosis, neutrophil infiltration, and infarct area, along with improved cardiac output. These findings confirm that NO_2_-FA can be functionalized within LNPs for targeted delivery when disrupted by UTC. Unlike prior systemic administration routes (intraperitoneal, subcutaneous, IV, or oral) this novel strategy enables localized, rapid drug concentration at sites of acute pathology, minimizing off-target effects and maximizing the biological stability of chemically reactive electrophilic NO_2_-FA.

Targeted delivery of NO_2_-FA to the heart using UTC+NO_2_-FA LNPs is an innovative approach that improves the precision and efficacy of vascular and cardioprotective therapy. UTC temporarily increases vascular permeability and promotes LNP + NO_2_-FA access to more remote cardiac tissue compartments at the site of LNP sonolysis. This strategy, successfully applied in both rodent and pig models, enhances cardiac drug delivery (Figure 1). UTC generates mechanical and thermal effects using high-frequency ultrasound, while LNPs protect NO_2_-FA from metabolism or alkylation and inactivation of the nitroalkene warhead by plasma nucleophiles. Concurrent systemic infusion and external ultrasound application enhances LNP accumulation in targeted myocardium. Higher concentrations in heart in comparison to remote site might be attributed to greater blood flow to myocardium and higher metabolic activity. UTC-mediated LNP delivery improves therapeutic uptake in infarcted or ischemic tissue 10, 38 delivering anti-fibrotic or pro-angiogenic agents, reducing fibrosis and improving cardiac function. Pig models, which closely mimic human cardiac physiology, demonstrate improved perfusion, reduced infarct size, and enhanced contractility with UTC-guided LNP delivery. This non-invasive technique allows spatially controlled drug targeting, minimizes off-target effects, and supports repeated use. LNPs can carry diverse therapeutic payloads, including small molecules, lipids, nucleic acids, and proteins.

UTC+NO_2_-FA LNPs showed significant cardioprotective effects, improving contractility, EF, and FS (Figures 2 and 4). Delivery of NO_2_-FA to the myocardium modulated redox signaling, reduced oxidative stress, and suppressed pro-inflammatory and pro-fibrotic pathways, mitigating cardiac damage and enhancing recovery. NO_2_-FA activates Nrf2-dependent expression of >100 antioxidant, cytoprotective, and tissue repair genes (e.g., HO-1, NQO-1, GCLM, glutathione transferases) 39, thereby protecting cardiomyocytes. By inhibiting TGF-β signaling and collagen deposition, NO_2_-FA reduces myocardial fibrosis, improves ventricular compliance and contractility. NO_2_-FA also enhances calcium cycling and mitochondrial function, boosting systolic performance and reducing apoptosis. In rodent IRI models, NO_2_-FA administration increased EF by 10-15%, reduced infarct size and fibrosis, and improved contractility 40-41. FS improved through reduced myocardial stiffness and enhanced systolic function. Similar benefits were observed in pig models, where NO_2_-FA decreased collagen deposition and fibroblast activation, improving diastolic function and further supporting EF and FS.

The electrophilic nature of NO_2_-FA enables reversible Michael addition with Cys thiolates on proteins and small molecules. This interaction inhibits pro-inflammatory enzymes having functionally significant hyper-reactive Cys residues, such as xanthine oxidoreductase 42, NADPH oxidases 43, cyclooxygenase, and 5-lipoxygenase 44. NO_2_-FA also modulate transcription factor function and signaling pathways, activating anti-inflammatory regulators like Nrf2 and PPARγ 45, 21, 46, while inhibiting TLR, NF-κB, JAK/STAT, STING and calcineurin 47 signaling pathways 17, 48-50. These pleiotropic actions induced by the multi-target electrophilic NO_2_-FA cargo creates an antioxidant and adaptive signaling environment that suppresses inflammatory cell activity, vaso-occlusion, and vascular remodeling. During ischemia-reperfusion injury (IRI), NO_2_-FA reduce the expression of multiple inflammatory genes. IRI promotes NF-κB translocation and increases TNF-α, which drives IL-6 and MCP-1 expression, amplifying inflammation, oxidative stress, and tissue damage, and worsening cardiovascular outcomes 51. UTC+NO_2_-FA LNP treatment significantly reduced inflammatory markers, myocardial injury biomarkers (troponin, LDH, CPK, myoglobin), and infarct size, benefits not observed with systemic NO_2_-FA administration (Figures 3 and 7).

UTC+NO_2_-FA LNPs reduced fibrosis and neutrophil infiltration primarily through anti-inflammatory and anti-fibrotic mechanisms (Figure 5). NO_2_-FA inhibits TGF-β signaling, a key mediator of fibrosis, thereby reducing cardiac fibroblast activation and collagen deposition. It also promotes macrophage polarization from pro-inflammatory M1 to anti-inflammatory M2 phenotypes, further suppressing fibrotic responses. By inhibiting pro-inflammatory cytokines (e.g., TNF-α, IL-6) and NF-κB activation, NO_2_-FA limit neutrophil infiltration and myocardial inflammation. In myocardial injury models (e.g., angiotensin II or IRI) 16, 52, NO_2_-FA significantly reduced collagen accumulation, neutrophil infiltration, and inflammation, improving EF and reducing infarct size.

UTC+NO_2_-FA LNPs also lowered expression of collagen 1, collagen 3, and α-SMA, as shown by immunohistochemistry (Figure 6), supporting its anti-fibrotic potential. NO_2_-FA suppresses TGF-β-induced fibroblast and myofibroblast activation, reducing ECM production. In fibrosis models, NO_2_-FA significantly decreased collagen 1/3 26 expression and α-SMA levels 41, leading to reduced fibrotic scarring and improved cardiac function. These effects were linked to decreased fibroblast activation and improved tissue remodeling.

UTC+NO_2_-FA LNPs reduced late gadolinium enhancement (LGE) by attenuating myocardial fibrosis and improved cardiac function, including stroke volume and cardiac output (Figure 8) 53, 54. These effects are driven by inhibition of TGF-β signaling and prevention of fibroblast-to-myofibroblast differentiation. NO_2_-FA has been shown to reduce fibrosis and enhance cardiac function in studies using cardiac magnetic resonance (CMR) imaging. In a murine dilated cardiomyopathy (DCM) model, NO_2_-FA reduced interstitial fibrosis, a major contributor to LGE 55, and significantly improved left ventricular systolic function by echocardiography. Although stroke volume and cardiac output were not directly reported, improved systolic function strongly suggests enhancement of these parameters. By modulating TGF-β signaling and blocking myofibroblast activation, NO_2_-FA reduces collagen deposition and fibrosis, improving cardiac performance and supporting better stroke volume and output.

Recent hydrogel-based strategies demonstrate integrated approaches for myocardial repair and regeneration. Wu et al. developed microenvironment-responsive hydrogels for stepwise infarct treatment 56, Bai et al. created conductive hydrogels enabling simultaneous monitoring and repair 57, and Liu et al. engineered stimulus-responsive multifunctional hydrogels supporting full-course myocardial regeneration 58. These studies highlight controlled, targeted, and multifunctional biomaterial therapies, complementing our UTC-facilitated focal delivery of NO₂-FA LNPs.

### Limitations and future directions

Despite the strengths of this study, some limitations warrant further consideration. Additional observation time points would improve characterization of short- and long-term myocardial tissue loading kinetics of NO₂-FA following ultrasound-targeted cavitation (UTC) and enable expanded temporal profiling of gene expression after ischemia–reperfusion injury. Protein-level validation of key molecular targets was not performed, and the absence of vascular-specific markers such as CD31 or von Willebrand factor limits interpretation of vascular remodeling and potential overlap in α-smooth muscle actin expression. Mechanistic analyses defining the sources of observed hemodynamic changes were beyond the scope of this project. Given the central role of nitric oxide signaling in NO₂-FA biology and ultrasound-mediated therapies, future studies could incorporate real-time, *in vivo* assessment of localized nitric oxide dynamics. Although NO₂-FA release kinetics were inferred from LNP shell composition and therapeutic outcomes, ultrasound-triggered versus endocytic release pathways were not directly compared. In addition, myocardial LNP spatial distribution, long-term biodistribution, and potential off-target accumulation were not evaluated. Interactions between LNPs and the myocardial microvascular endothelium were not directly examined. While prior studies suggest preserved microvascular architecture after UTC+NO₂-FA LNP therapy, future investigations using high-speed intravital microscopy could clarify endothelial interactions, *in vivo* LNP size dynamics, and transport mechanisms. Other issues include the absence of cavitation-controlled ultrasound groups, incomplete profiling of macrophage subsets, lack of advanced structural characterization such as cryo-electron microscopy, absence of diastolic functional assessment, and lack of long-term efficacy studies in female or aged animals.

Future work and pending research support applications can further extend the present study by incorporating protein-level assays, vascular markers, and fluorescently or radiolabeled NO₂-FA or LNP tracers to directly evaluate release mechanisms, myocardial and systemic distribution, retention, pharmacokinetics, and off-target deposition. Expanded molecular analyses, including oxidative stress markers, macrophage phenotyping, and transcriptomic or proteomic approaches, will further define pathways underlying NO₂-FA-mediated cardioprotection. Optimization of ultrasound parameters will aim to maximize delivery efficiency while ensuring safety. Translation to larger animals and humans will require additional refinement, including long-term follow-up (1-3 months), assessment of repeated ultrasound exposure, and studies in ovariectomized and aged models. Efficacy testing in porcine models of myocardial ischemia or heart failure will improve clinical relevance. Finally, clinical translation will require scalable NO₂-FA LNP manufacturing, formulation optimization, further stability and toxicology evaluation via IND-enabling studies to assure safe and effective dosing and delivery strategies.

## Conclusion

In summary, we successfully established robust rat models of myocardial ischemia-reperfusion injury (MIRI) and myocardial fibrosis and demonstrated that UTC-mediated delivery of NO₂-FA LNPs markedly improved cardiac function. In MIRI, UTC+NO₂-FA LNPs enhanced myocardial contractility, ejection fraction, and fractional shortening, while reducing infarct size, pro-inflammatory gene expression, and myocardial injury markers, outperforming both intravenous NO₂-FA and untreated controls. In the myocardial fibrosis model, UTC+NO₂-FA LNPs produced the most pronounced improvements in myocardial contractility, left ventricular fractional area change, and wall motion score index. Treatment also attenuated fibrosis, neutrophil infiltration, collagen I/III and α-SMA deposition, and late gadolinium enhancement, while enhancing stroke volume, cardiac output, and longitudinal strain. Collectively, these findings highlight the novel cardioprotective and antifibrotic efficacy of UTC+NO₂-FA LNPs, supporting their potential as a targeted therapeutic strategy for ischemic heart disease.

### Statistical analysis

All quantitative data were analyzed in GraphPad Prism. Data are presented as the mean ± SEM. One-way analysis of variance (ANOVA) followed by Tukey’s multiple comparisons test was used to detect significance compared with the UTC+NO_2_-FA LNPs. In wall motion score index and fractional area change, multiple unpaired t tests were performed to detect significance between pre-treatment and post-treatment of each group and discoveries are mentioned as q value where applicable. In all cases, *p < 0.05, **p < 0.01, and ***p < 0.001.

### Use of artificial intelligence tools

In accordance with COPE guidelines and the TITAN Guideline Checklist (2025), the authors report limited use of an artificial intelligence-based language model (ChatGPT, OpenAI). The tool was used exclusively for editorial assistance, including grammar correction, sentence restructuring, and clarity enhancement of narrative text in non-methodological sections of the manuscript (e.g., Introduction and Discussion).

The AI tool was not used for experimental design, animal procedures, data acquisition, data processing, statistical analysis, image generation or modification, result interpretation, or manuscript conclusions. All experimental work, data analysis, and figure preparation were performed by the authors, who retain full responsibility for the scientific content and conclusions presented.

## Supplementary Material

Supplementary methods.

## Abbreviations

AI: artificial intelligence
ABC: avidin-biotin complex
ANOVA: analysis of variance
BSA: bovine serum albumin
cTn-I: cardiac troponin I
CMR: cardiac magnetic resonance imaging
CPK: creatine phosphokinase
DAB: 3,3′-diaminobenzidine
DLS: dynamic light scattering
ECV: extracellular volume
EF: ejection fraction
ELISA: enzyme-linked immunosorbent assay
FFPE: formalin-fixed paraffin-embedded
FS: fractional shortening
Gd: gadolinium
GAPDH: glyceraldehyde-3-phosphate dehydrogenase
H&E: hematoxylin and eosin
HF: heart failure
HPLC-ESI-MS/MS: high-performance liquid chromatography-electrospray ionization–tandem mass spectrometry
IACUC: Institutional Animal Care and Use Committee
IF: immunofluorescence
IHC: immunohistochemistry
IRI: ischemia–reperfusion injury
LAD: left anterior descending coronary artery
LDH: lactate dehydrogenase
LGE: late gadolinium enhancement
LNP: lipid nanoparticle
LV: left ventricle
MI: myocardial infarction
MCP-1: monocyte chemoattractant protein-1
MIRI: myocardial ischemia–reperfusion injury
MRI: magnetic resonance imaging
MRM: multiple reaction monitoring
NF-κB: nuclear factor kappa B
NO2-FA: nitro-fatty acid
NO2-FA-LNP: nitro-fatty acid–loaded lipid nanoparticle
PBS: phosphate-buffered saline
PCR: polymerase chain reaction
PEG: polyethylene glycol
SEM: standard error of the mean
ST2: suppression of tumorigenicity 2
SV: stroke volume
TEM: transmission electron microscopy
TNF-α: tumor necrosis factor alpha
TTC: triphenyl tetrazolium chloride
US: ultrasound
UTC: ultrasound-targeted cavitation
VFA: variable flip angle
