# Supplementary material for: Ultrasound targeted fatty acid nitroalkene-containing lipid nanoparticles attenuate inflammation and reverse myocardial fibrosis
Source: Theranostics. 2026 May 29;16(13):7088–107. doi: 10.7150/thno.126503 (PMC13294811; doi:10.7150/thno.126503)
Supplement: Supplementary file 1 — Supplementary methods. [file thnov16p7088s1.pdf]

Nitro-fatty acid LNP (NO_2-FA-LNP) Synthesis and Characterization

NO_2-FA-loaded lipid nanoparticles (NO_2-FA-LNPs) were formulated using 1,2-distearoyl-sn-glycero-3-phosphocholine, 1,2-distearoyl-sn-glycero-3-phosphoethanolamine-N-(methoxy(polyethylene glycol)-2000) (Avanti Polar Lipids Inc., Alabaster, AL), polyoxyethylene-40 stearate (Sigma Aldrich, St. Louis, MO), and 10-nitro-octadec-9-enoic acid (NO_2-FA). The lipid constituents in powder form were accurately weighed and mixed in a glass vial according to a molar ratio of 14.04:1.85:16.74:14.13. Propylene glycol, glycerol, and phosphate-buffered saline were subsequently added, and the mixture was incubated in a 56°C water bath for 20 minutes to ensure uniform dispersion.

Following incubation, the formulation was aliquoted (1 mL per vial) into screw-cap clear vials equipped with polypropylene perforated caps and silicone septa. The vial headspace was replaced with perfluorobutane gas (FluoroMed, LP, Round Rock, TX, USA). Prepared vials were stored under refrigeration and mechanically activated immediately prior to use with a Vialmix® device (Lantheus Medical Imaging, Inc., North Billerica, MA) to generate NO_2-FA-LNPs. Before administration, activated particles were washed to eliminate unincorporated NO_2-FA.

The synthesized NO_2-FA-LNPs were subsequently evaluated for particle concentration, size distribution, and NO_2-FA loading efficiency. Particle concentration and size distribution were measured using a Coulter Counter (Multisizer 3, Beckman Coulter, Fullerton, CA, USA), which provides direct measurement of volume-based particle size distributions and is particularly suitable for micron and submicron lipid-based particles. Measurements were conducted under standardized electrolyte and dilution conditions to maintain reproducibility. Although dynamic light scattering (DLS) and transmission electron microscopy (TEM) were not performed, Coulter counter analysis offered reliable quantitative evaluation of particle concentration and size distribution relevant to ultrasound-mediated cavitation behavior.

Encapsulation efficiency of NO_2-FA was measured by HPLC-ESI-MS/MS after removal of free (unloaded) NO_2-FA through washing. Incorporated NO_2-FA was calculated by subtracting subnatant NO_2-FA from the total NO_2-FA concentration measured in the LNP suspension, thereby enabling selective quantification of LNP-associated NO_2-FA. Separation was achieved using an analytical C18 Luna column (2 × 20 mm, 5 µm; Phenomenex) operated at a flow rate of 0.7 mL/min. A gradient elution was employed with aqueous 0.1% acetic acid (solvent A) and acetonitrile containing 0.1% acetic acid (solvent B). The gradient increased from 35% to 100% solvent B over 0-3 minutes, was maintained at 100% solvent B from 3-4 minutes, and was then returned to the initial conditions with a 1-minute re-equilibration period.

NO_2-FA was detected in negative ion mode using a QTRAP 6500+ triple quadrupole mass spectrometer (Sciex, Framingham, MA). Instrument settings included a declustering potential of −50 V, collision energy of −42 eV, source temperature of 650°C, and monitoring of the multiple reaction monitoring (MRM) transition 326.2/46. Quantitative analysis was performed by stable isotopic dilution using a calibration curve constructed with $[^{15}N]O_2$-[d4]OA as the internal standard (MRM 331.2/47). The concentration of NO_2-FA incorporated into NO_2-FA-LNPs was determined by subtracting free NO_2-FA from the total measured NO_2-FA in solution.

Analysis of NO_2-FA in NO_2-FA LNP and Tissue Samples

To quantify NO_2-FA in nanoparticle formulations, both the NO_2-FA-LNP suspension in saline and the corresponding saline supernatant were diluted with acetonitrile. Each preparation was spiked with 1.5 pmol of $[^{15}N]O_2$-[d4]OA internal standard and analyzed by HPLC-MS/MS. The amount of NO_2-FA incorporated into LNPs was calculated by subtracting the NO_2-FA concentration detected in the supernatant from the total NO_2-FA measured in the LNP suspension.

For hindlimb delivery studies, tissue specimens were collected from the treated gastrocnemius muscle as well as from a remote control site (quadriceps femoris muscle) on the same hindlimb. Samples were immediately snap-frozen in liquid nitrogen and stored at –80°C until processing. In the myocardial ischemia-reperfusion injury (MIRI) and fibrosis experiments, tissue was obtained from the treated myocardial left ventricle and from the ipsilateral quadriceps femoris muscle as a remote control.

Approximately 200 mg of frozen tissue was thawed and homogenized in 50 mM phosphate-buffered saline (pH 7.4) using a bullet blender (Next Advance™). A 500 µL portion of the homogenate was spiked with 1 pmol of $[^{15}N]O_2$-[d4]OA internal standard. Lipid extraction was carried out by adding 1.25 mL of hexane–isopropanol–1M formic acid (30:20:2, vol/vol/vol), vortexing, and subsequently adding 1.25 mL hexane. After mixing and centrifugation, the upper organic layer containing NO_2-FA was collected, dried under nitrogen, and reconstituted in 100 µL acetonitrile for HPLC-MS/MS analysis. The differential accumulation of NO_2-FA between treated and remote tissues was determined by subtracting their respective concentrations.

Animals

Male Wistar rats (Envigo, Indianapolis, IN, USA) weighing 250–300 g were utilized for all rodent studies. All experimental procedures involving rats were performed in compliance with institutional animal care and use guidelines (IACUC protocol #22112218).

Two female Rapacz Familial Hypercholesterolemic swine (60–70 kg; University of Wisconsin), one receiving IV NO_2-FA infusion and one receiving UTC+NO_2-FA LNP infusion, were utilized to evaluate targeted cardiac delivery. Porcine experiments were performed under IACUC protocol #20128306 and are reported in accordance with ARRIVE recommendations where applicable.

Animals were assigned to experimental groups based on study design considerations aimed at feasibility and proof-of-concept evaluation. Randomization and investigator blinding were not incorporated in this preliminary phase. All surgical procedures, treatments, and analytical methods were conducted according to standardized protocols by experienced personnel to minimize experimental variability. Future investigations will incorporate randomization and blinding to further strengthen experimental rigor.

Rat Hindlimb Targeted Drug Delivery

The healthy hindlimb model was established to evaluate tissue delivery of NO_2-FA and comprised two treatment arms: intravenous (IV) NO_2-FA and UTC+NO_2-FA LNP. The administered NO_2-

FA dose (260 μg) was maintained consistently across groups and represented the NO_2-FA content contained within 3 × 10^9 NO_2-FA-LNP. All nanoparticle treatments, irrespective of loading status, consisted of 3 × 10^9 particles per administration. Infusions (IV NO_2-FA or NO_2-FA-LNP) were performed via femoral vein cannulation at a rate of 3 mL/hr.

For UTC therapy, therapeutic ultrasound was delivered using a single-element transducer operating at 1 MHz (A303S, 0.5 inch, Olympus NDT, Waltham, MA), driven by an arbitrary waveform generator (AFG3252, Tektronix, Beaverton, OR) and amplified with a radio frequency power amplifier (800A3B, Amplifier Research, Souderton, PA). The acoustic output was calibrated using a 200-μm capsule hydrophone (HGL-0200, Onda Corp, Sunnyvale, CA, USA).

The transducer was oriented vertically over the gastrocnemius muscle of the left hindlimb while the rat was positioned in right lateral decubitus, approximately 1.5 cm from the limb surface. Ultrasound gel was applied to ensure effective acoustic coupling. Pulsed ultrasound (center frequency 1 MHz; peak negative acoustic pressure 1.5 MPa; pulse duration 5 ms) was delivered every 3 seconds for a total of 10 minutes to allow replenishment of LNPs within the insonified region.

Proper positioning of the transducer over the gastrocnemius muscle was confirmed by direct visualization and by observing a single LNP destruction pulse within the imaging field prior to treatment initiation. Physiologic parameters, including heart rate, respiratory rate, and oxygen saturation, were continuously monitored throughout the procedure.

Following two consecutive 10-minute treatment sessions, animals were euthanized with an overdose of isoflurane, followed by cardiac excision. Tissue samples were harvested from the UTC-treated gastrocnemius muscle, guided by ultrasound localization markers. Samples from the ipsilateral quadriceps femoris muscle were collected as remote controls that received systemic infusion without targeted ultrasound exposure. All tissues were immediately snap-frozen in liquid nitrogen and stored at –80°C for further analysis.

Porcine Myocardial Drug Delivery

Pigs were anesthetized, intubated, and mechanically ventilated throughout the procedure. Physiologic parameters were monitored at regular intervals. Separate animals received either IV NO_2-FA or NO_2-FA-LNP via ear vein infusion for 20 minutes. The administered dose of NO_2-FA was maintained at 5200 μg per pig, irrespective of formulation.

During NO_2-FA-LNP administration, therapeutic ultrasound was applied to the myocardium spanning from the base to the apex of the left ventricle using a modified EpiQ imaging system equipped with an S5-1 probe (Philips Healthcare). The ultrasound settings included a center frequency of 1.6 MHz, pulse duration of 1 ms, and a peak negative acoustic pressure of 1.3 MPa. Infusions were maintained at a rate of 3 mL/min over a 20-minute period.

For IV NO_2-FA administration, 5200 μg NO_2-FA was diluted in 60 mL saline and infused at 3 mL/min for 20 minutes using a syringe pump. For NO_2-FA-LNP treatment, 20 mL of NO_2-FA-

LNP suspension containing 5200 µg NO_2-FA was diluted with 40 mL saline (total volume 60 mL) and administered at the same infusion rate and duration.

After completion of the treatment protocol, animals were euthanized via potassium chloride (KCl) injection, and the heart was promptly excised. Tissue specimens were obtained from the UTC-treated left ventricular myocardium. In addition, quadriceps femoris muscle from the ipsilateral hindlimb was collected as a remote-control tissue that received systemic infusion without targeted ultrasound exposure. All samples were immediately snap-frozen in liquid nitrogen and stored at –80°C for subsequent analysis.

Development of Acute Myocardial Ischemia-Reperfusion Injury Model

Male rats aged two months (275 ± 25 g) were anesthetized with 2.5% inhaled isoflurane and mechanically ventilated using a RoVent® Jr. small animal ventilator (Kent Scientific Corporation, Torrington, CT, USA). The jugular vein was cannulated to facilitate administration of NO_2-FA-LNP or intravenous (IV) NO_2-FA. Following chest shaving, baseline echocardiography was performed to assess left ventricular function.

A left thoracotomy was carried out by entering the thoracic cavity between the fourth and fifth ribs, and a surgical retractor was positioned to maintain adequate exposure. The pericardium was gently dissected to allow visualization of the heart, and the left anterior descending (LAD) coronary artery was identified. By carefully retracting the right ribs, the heart was partially exteriorized, and the LAD was ligated with a 6-0 surgical suture to induce ischemia. A short segment of flexible rubber tubing was positioned beneath the ligature to allow subsequent release of the suture without causing myocardial injury.

Myocardial ischemia was confirmed by immediate pallor of the left ventricular myocardium distal to the ligation site and by regional wall motion abnormalities on echocardiography. The heart was repositioned within the thoracic cavity, which was temporarily closed. Echocardiography was repeated to assess left ventricular function during ischemia.

After 45 minutes of LAD occlusion, the thoracic cavity was reopened, the heart was gently lifted, and the ligature was released to initiate reperfusion. The heart was repositioned within the thoracic cavity, and the chest was subsequently closed. Reperfusion was allowed for 20 minutes. Successful reperfusion was confirmed by restoration of myocardial color and improvement in contractility following release of the ligature.

Four experimental groups were included: sham (no LAD ligation), control (LAD ligation without treatment), NO_2-FA-LNP infusion combined with UTC treatment, and intravenous IV NO_2-FA (formulated in 0.2% ethanol) infusion. Both NO_2-FA-LNP and IV NO_2-FA were delivered over 15 minutes using a syringe pump operating at 4 mL/h. During administration, the syringe was gently rotated to ensure continuous suspension of the LNPs.

Following treatment, animals were maintained under light anesthesia (1%) for 2 hours. Echocardiography was performed again at 2 hours post-treatment. The thoracic cavity was

reopened, blood was collected via left ventricular puncture, and serum was isolated and stored at –80°C for subsequent analysis. Animals were then euthanized.

Tissue Processing for Acute Myocardial Ischemia-Reperfusion Injury (MIRI) Study

Cardiac tissue was preserved using two different methods depending on the intended downstream analysis. For molecular studies, including polymerase chain reaction (PCR), hearts were excised, rinsed thoroughly with ice-cold PBS to remove residual blood, divided into three equal sections, and stored at –80°C until further processing.

For histopathological assessment, including Masson's Trichrome staining, harvested hearts were rinsed with ice-cold PBS and fixed in formalin. Tissues were then processed into formalin-fixed paraffin-embedded (FFPE) blocks. Sections were stained using a standard Trichrome protocol (Histowiz, Long Island City, NY), followed by counterstaining with Weigert's Hematoxylin. Slides were dehydrated and coverslipped using a TissueTek-Prisma and Coverslipper system (Sakura). Whole-slide images were acquired at 40× magnification with an Aperio AT2 scanner (Leica Biosystems).

For enzyme-linked immunosorbent assays (ELISAs), serum samples obtained from each treatment group were used to quantify relevant biomarkers.

Ultrasound-Targeted Cavitation Therapy, Echocardiography, and Image Processing for MIRI

UTC therapy was administered using a single-element transducer with a center frequency of 1 MHz (A303S, 0.5 inch, Olympus NDT, Waltham, MA). The transducer was powered by an arbitrary waveform generator coupled to a radio frequency power amplifier (800A3B, Amplifier Research, Souderton, PA). System output was acoustically calibrated using a 200-μm capsule hydrophone (HGL-0200, Onda Corp, Sunnyvale, CA, USA) to confirm the delivered pressure parameters.

The treatment transducer was positioned vertically over the left hemithorax with the rat maintained in the supine position, approximately 1.5 cm from the chest surface. Ultrasound gel was applied to ensure proper acoustic coupling. Rapid short ultrasound pulses (center frequency 1 MHz; peak negative acoustic pressure 0.5 MPa; pulse duration 10 μs ON and 90 μs OFF; 50 pulses per burst; bursts repeated every 0.5 seconds) were delivered for a total treatment duration of 15 minutes to allow replenishment of LNPs within the myocardial microcirculation.

Ultrasound parameters were selected based on previous studies demonstrating efficient acoustic cavitation and drug delivery using lipid-based contrast agents and gas-filled nanoparticles at similar frequencies and pressures. Calibration with a capsule hydrophone ensured accurate delivery of acoustic pressure. Although cavitation threshold measurements were not directly performed, visualization of LNP destruction within the ultrasound field served as a functional confirmation of cavitation activity.

Echocardiographic assessments were performed at three time points: baseline, during ischemia, and 2 hours after treatment, using the Vevo 2100 imaging system (FUJIFILM VisualSonics Inc., Bothell, WA). Short-axis B-mode images were acquired to evaluate left ventricular wall motion and to identify anteroseptal wall abnormalities resulting from LAD ligation. M-mode recordings were used to assess left ventricular contractility and to calculate left ventricular ejection fraction (EF %) and fractional shortening (FS %). The transducer operated at 21 MHz with a frame rate of 12. Gain settings were adjusted individually to optimize image quality.

Image analysis was conducted using Vevo LAB software, which provides tools for visualization and quantitative assessment of myocardial ischemia-reperfusion injury. Using the "LV trace" function, at least three complete cardiac cycles were delineated for each measurement, enabling calculation of EF (%), FS (%), left ventricular mass, and additional functional parameters.

Inflammatory Response Analysis

Total RNA was isolated from approximately 50 mg of myocardial tissue using TRIzol reagent (Life Technologies) following the manufacturer's protocol. RNA concentration and purity were assessed with a SpectraMax QuickDrop Micro-Volume spectrophotometer (Molecular Devices, San Jose, CA, USA). Complementary DNA (cDNA) was generated from 1 μg of total RNA using the iScript cDNA Synthesis Kit (Bio-Rad, Hercules, CA, USA).

Quantitative RT-PCR was performed using a CFX Connect Real-Time System (Bio-Rad) with SsoAdvanced Universal SYBR Green Supermix (Bio-Rad). Relative gene expression was normalized to GAPDH as the internal control. The inflammatory genes analyzed included monocyte chemotactic protein 1 (MCP-1), tumor necrosis factor alpha (TNF-α), interleukin-6 (IL-6), and nuclear factor kappa-light-chain-enhancer of activated B cells (NF-κB). Primer sequences used for amplification were as follows:

MCP-1 (For) 5'- CTGACCCCAATAAGGAATG-3'

MCP-1 (Rev) 5'- TGAGGTGGTTGTGGAAAAGA-3'

TNF-α (For) 5'- CTCTTCTCATTCCCGCTCGT-3'

TNF-α (Rev) 5'- GGGAGCCCATTTGGGAACTT-3'

IL-6 (For) 5'- CCAGTTGCCTTCTTGGGACT-3'

IL-6 (Rev) 5'- TCTGACAGTGCATCATCGCT-3'

NF-κB (For) 5'- ACGATCTGTTTCCCCTCATCT-3'

NF-κB (Rev) 5'- TGGGTGCGTCTTAGTGGTATC-3'

GAPDH (For) 5'- AAACCCATCACCATCTTCCA-3'

GAPDH (Rev) 5’- GTGGTTCACACCCATCACAA-3’

Quantitative Sandwich ELISA

Serum levels of cardiac troponin (cTn-I), creatinine phosphokinase (CPK), myoglobin, and lactate dehydrogenase (LDH) were quantified using commercially available ELISA kits (MyBioSource, San Diego, CA, USA) in accordance with the manufacturer’s instructions. Previously collected and stored serum samples were used for these analyses. Absorbance was measured at 450 nm with a DTX 880 Multimode Detector (Beckman Coulter, Brea, CA, USA). The resulting optical density (OD) values were normalized to total protein content as determined by the Bradford assay. All samples were analyzed in duplicate. ELISAs were performed in both the MIRI and fibrosis experiments.

Rat Myocardial Fibrosis Model

A rat model of myocardial fibrosis was developed to assess the anti-inflammatory and anti-fibrotic efficacy of UTC+NO_2-FA-LNP. Following a protocol similar to the IRI model, the LAD coronary artery was occluded for 60 minutes. The ligature was then released to allow reperfusion, the thoracic cavity was sutured closed, and animals were permitted to recover from anesthesia. Rats were housed individually for 28 days to allow progression of myocardial fibrosis.

Treatment was initiated on day 28 following ischemia-reperfusion and continued for two weeks, with administration on alternate days (total of 7 treatments). At the end of the treatment period, echocardiography was performed, serum samples were collected, and animals were euthanized. Hearts were preserved either in formalin for histological analysis or stored at –80°C for molecular studies.

Myocardial fibrosis was assessed by Masson’s trichrome staining, and the fibrotic region was quantified as a percentage of the total myocardial area. Quantification was performed on mid-ventricular transverse sections to ensure consistent anatomical comparison across animals. Analyses included infarct, peri-infarct, and remote myocardial regions where applicable.

Echocardiography and Image Processing for Fibrosis Experiment

The UTC treatment settings used in the fibrosis study were identical to those employed in the MIRI experiment. Echocardiographic evaluations were conducted at three time points: baseline, during ischemia, and following the 14-day treatment period. Imaging was performed with a clinical ultrasound system (Siemens Sequoia, 15L8 probe) to assess segmental wall motion using both B-mode and M-mode modalities.

Short-axis B-mode images of the left ventricle were acquired to assess the impact of fibrosis on the anteroseptal wall, and a left ventricular wall motion score index was calculated. M-mode recordings were also obtained to evaluate anterior wall contractility and to calculate left ventricular fractional area change.

Histopathological Analysis

H&E Staining

At the end of the study, rat hearts were harvested and fixed in 10% neutral buffered formalin. The tissues were then processed into formalin-fixed paraffin-embedded (FFPE) blocks. Sections were stained according to a standard hematoxylin and eosin (H&E) protocol (Histowiz, Long Island City, NY). Following staining, slides were dehydrated and coverslipped using a TissueTek-Prisma and Coverslipper system (Sakura). Whole-slide scans were acquired at 40× magnification using an Aperio AT2 scanner (Leica Biosystems).

Masson's Trichrome Staining

Formalin-fixed paraffin-embedded (FFPE) sections were additionally stained with Masson's Trichrome according to a standard protocol (Histowiz, Long Island City, NY). After staining, sections were counterstained with Weigert's Hematoxylin, dehydrated, and coverslipped using a TissueTek-Prisma and Coverslipper system (Sakura). Whole-slide images were obtained at 40× magnification using an Aperio AT2 scanner (Leica Biosystems).

Immunohistochemistry (IHC) Analysis

After euthanasia, hearts were promptly removed and rinsed in ice-cold phosphate-buffered saline (PBS) to eliminate residual blood. Tissues were immersed in 10% neutral buffered formalin and fixed for 24–48 hours at 4°C. Following fixation, samples were processed through graded ethanol (70%, 80%, 95%, and 100%) for dehydration, cleared in xylene, and embedded in paraffin using an automated tissue processor.

Paraffin blocks were sectioned at 4-5 µm thickness using a microtome, and sections were mounted on positively charged glass slides. Slides were dried overnight at 37°C to promote tissue adherence. Deparaffinization was achieved with two 5-minute xylene washes, followed by rehydration through descending ethanol concentrations (100%, 95%, 80%, and 70%) and a final rinse in distilled water.

Antigen retrieval was performed using Tris-EDTA buffer (pH 9.0) for collagen I and collagen III, and citrate buffer (pH 6.0) for α-SMA. Slides were heated in a microwave for antigen unmasking, allowed to cool at room temperature for 20–30 minutes, and then washed in PBS (pH 7.4).

To inhibit endogenous peroxidase activity, sections were incubated in 3% hydrogen peroxide prepared in methanol for 10 minutes at room temperature, followed by PBS washes. Non-specific binding sites were blocked by incubating the sections in 5% normal serum diluted in PBS for 1 hour at room temperature.

Primary antibodies were prepared in PBS containing 1% bovine serum albumin (BSA) and applied at the following dilutions: rabbit polyclonal anti-collagen I (1:200), rabbit polyclonal anti-collagen III (1:200), and mouse monoclonal anti-α-SMA (1:100). Slides were incubated overnight at 4°C in a humidified chamber. Negative control sections were processed in parallel with omission of the primary antibody and substitution with PBS.

After PBS washing, species-specific biotinylated secondary antibodies (1:200 dilution in PBS) were applied for 1 hour at room temperature. Slides were rinsed and subsequently incubated with avidin-biotin-peroxidase complex (ABC) reagent for 30 minutes. Visualization was achieved using 3,3’-diaminobenzidine (DAB) substrate for 5–10 minutes, with microscopic monitoring to avoid excessive staining. The reaction was terminated by rinsing in distilled water.

All stained slides were scanned at 40× magnification using an Aperio AT2 digital slide scanner (Leica Biosystems).

Evans Blue / TTC Staining

To delineate infarcted and fibrotic regions within the left ventricle, Evans blue and triphenyl tetrazolium chloride (TTC) staining was performed. Immediately prior to euthanasia, the left anterior descending (LAD) coronary artery was re-ligated at the same site used previously to induce ischemia. Evans blue dye (1.5%, 1.0 mL) was administered via jugular vein cannulation and allowed to circulate for three cardiac cycles. Potassium chloride (KCl) was then injected to arrest the heart in systole.

The heart was removed, washed, and cut into transverse slices approximately 2 mm in thickness. The slices were then incubated in 1% TTC solution in a petri dish at 37°C for 15 minutes. Stained sections were imaged using a Leica microscope, and both the area at risk and the ischemic area (%) were quantified with FIJI software.

In Vivo Cardiac MRI (CMR)

In vivo cardiac MRI examinations were performed using a Bruker BioSpec 70/30 USR scanner (Bruker BioSpin MRI, Billerica, MA) operating at 7 Tesla and fitted with a 35-mm quadrature volume coil for both transmission and reception. During imaging, animals were maintained under 1–2% inhaled isoflurane administered via a nose cone. Core body temperature was regulated at 36.2 ± 0.8°C, and respiration was continuously monitored with an MRI-compatible pneumatic sensor and pressure transducer (SA Instruments, Inc., Model 1025).

Cine CMR

Cine CMR imaging was performed using a fast, free-breathing, non-gated protocol to acquire multi-slice short-axis (SA), long-axis two-chamber (LA2C), and long-axis four-chamber (LA4C) views. Imaging settings were as follows: field of view (FOV) 5 × 5 cm, slice thickness (SLTH) 1.5 mm for SA and 2 mm for LA, matrix 256 × 256, in-plane spatial resolution 0.195 mm, echo time (TE) 3.059 ms, repetition time (TR) 5.653 ms, flip angle (FA) 10°, number of repetitions (NR) 250, 20 cardiac phases, and a total acquisition time (TT) of 3 minutes 2 seconds per sequence.

Left ventricular volume, ejection fraction (EF), and stroke volume (SV) were quantified using Circle cvi42 software (Circle Cardiovascular Imaging, Inc.) to assess systolic function. Myocardial strain analysis was also performed, with circumferential, radial, and longitudinal strain calculated across the cardiac cycle to evaluate ventricular deformation.

Contrast-Enhanced CMR

Myocardial fibrosis was assessed by calculating extracellular volume (ECV) using gadolinium (Gd)-enhanced imaging. Baseline (pre-contrast) T1 mapping was obtained prior to contrast administration. Gadobenate dimeglumine (MultiHance®, Bracco Diagnostics, Inc.) was administered as a single bolus at a dose of 0.2 mmol/kg body weight via a subcutaneous catheter (McKesson IV Catheter 25G × 0.75", Product No. 854664). Approximately 20 minutes after injection, steady-state distribution of Gd within the blood pool and myocardium was achieved. Post-contrast T1 mapping was subsequently acquired, followed by late gadolinium enhancement (LGE) imaging.

Jugular vein T1 mapping was acquired immediately before and after myocardial T1 mapping to determine blood T1 values. Cine CMR imaging was completed during the waiting period prior to reaching Gd steady state.

Myocardial T1 Mapping

Myocardial T1 values were quantified using an established variable flip angle (VFA) method [55]. Free-breathing, non-gated T1 mapping with four flip angles was performed using the following parameters: FOV 5 × 5 cm, matrix 256 × 256, in-plane resolution 0.195 mm, SLTH 1.5 mm, flip angles 3°, 19°, 22°, and 28°, TE 3.059 ms, TR 200 ms, NR 10, and TT 2 minutes 26 seconds.

Blood T1 Mapping (Jugular Vein)

Blood T1 mapping was performed in the jugular veins (JV) both before and after myocardial T1 acquisition. Free-breathing, non-gated T1 mapping with four flip angles was acquired using the following parameters: FOV 4.5 × 4.5 cm, matrix 128 × 128, in-plane resolution 0.352 mm, SLTH 2 mm, flip angles 3°, 19°, 22°, and 28°, TE 2.349 ms, TR 77.158 ms, NR 10, and TT 49 seconds. Imaging planes were aligned with the jugular veins to fully include the vessel lumen and avoid through-plane flow artifacts.

Extracellular Volume (ECV) Calculation

Myocardial ECV was calculated using the equation:

$$(1 - \text{hematocrit}) \times (\Delta R1\ \text{myocardium} / \Delta R1\ \text{blood})$$

where R1 represents the longitudinal relaxation rate (1/T1). Myocardial R1 values were obtained from left ventricular myocardium, and blood R1 values were derived from jugular vein measurements. ΔR1 corresponds to the difference in R1 values before and after gadolinium administration.

Hematocrit Measurement

Blood was obtained from the facial vein using a 5.5-mm sterile blood lancet (Goldenrod Animal Lancet). The samples were loaded into micro-hematocrit capillary tubes, sealed with clay, and

centrifuged for 5 minutes in a micro-hematocrit centrifuge to separate plasma from cellular elements. Hematocrit was determined by calculating the proportion of packed red blood cell volume relative to the total blood volume.

Statistical Analysis

All quantitative data were analyzed using GraphPad™ Prism software. Results are presented as mean ± SEM. Group comparisons were performed using one-way analysis of variance (ANOVA) followed by Tukey's post hoc multiple comparisons test, with statistical significance assessed relative to the UTC+NO_2-FA LNP group where specified.

For wall motion score index and fractional area change, multiple unpaired t-tests were used to evaluate differences between pre-treatment and post-treatment measurements within each group, and corresponding findings are reported as q values when applicable. Statistical significance thresholds were set at *$p < 0.05$, **$p < 0.01$, and ***$p < 0.001$.

Use of Artificial Intelligence Tools

Consistent with COPE guidelines and the TITAN Guideline Checklist (2025), the authors disclose limited use of an artificial intelligence-based language model (ChatGPT, OpenAI). The tool was utilized solely for editorial support, including grammatical correction, sentence refinement, and improvement of clarity in narrative, non-methodological sections of the manuscript (e.g., Introduction and Discussion).

Artificial intelligence was not employed for experimental design, animal experimentation, data collection, data processing, statistical evaluation, image creation or alteration, interpretation of findings, or development of manuscript conclusions. All experiments, analyses, and figure preparation were conducted by the authors, who assume full responsibility for the integrity, accuracy, and scientific conclusions of the work.
